# Discovering the Potential of Natural Antioxidants in Age-Related Macular Degeneration: A Review

**DOI:** 10.3390/ph15010101

**Published:** 2022-01-14

**Authors:** Kah-Hui Wong, Hui-Yin Nam, Sze-Yuen Lew, Murali Naidu, Pamela David, Tengku Ain Kamalden, Siti Nurma Hanim Hadie, Lee-Wei Lim

**Affiliations:** 1 Department of Anatomy, Faculty of Medicine, Universiti Malaya, Kuala Lumpur 50603, Malaysia; szeyuenlew@gmail.com (S.-Y.L.); murali_naidu@um.edu.my (M.N.); rosiepamela@um.edu.my (P.D.); 2 Neuromodulation Laboratory, School of Biomedical Sciences, Li Ka Shing Faculty of Medicine, The University of Hong Kong, 21 Sassoon Road, Pokfulam, Hong Kong, China; 3 Tissue Engineering Group, Department of Orthopaedic Surgery (NOCERAL), Faculty of Medicine, Universiti Malaya, Kuala Lumpur 50603, Malaysia; huiyin26@yahoo.com; 4 UM Eye Research Centre, Department of Ophthalmology, Faculty of Medicine, Universiti Malaya, Kuala Lumpur 50603, Malaysia; taftkamalden@um.edu.my; 5 Department of Anatomy, School of Medical Sciences, Health Campus, Universiti Sains Malaysia, Kubang Kerian, Kota Bharu 16150, Kelantan, Malaysia; snurma@usm.my

**Keywords:** age-related macular degeneration, oxidative damage, retina, angiogenesis, antioxidants

## Abstract

Age-related macular degeneration (AMD) is a multifactorial disease associated with anatomical changes in the inner retina. Despite tremendous advances in clinical care, there is currently no cure for AMD. This review aims to evaluate the published literature on the therapeutic roles of natural antioxidants in AMD. A literature search of PubMed, Web of Science and Google Scholar for peer-reviewed articles published between 1 January 2011 and 31 October 2021 was undertaken. A total of 82 preclinical and 18 clinical studies were eligible for inclusion in this review. We identified active compounds, carotenoids, extracts and polysaccharides, flavonoids, formulations, vitamins and whole foods with potential therapeutic roles in AMD. We evaluated the integral cellular signaling pathways including the activation of antioxidant pathways and angiogenesis pathways orchestrating their mode of action. In conclusion, we examined the therapeutic roles of natural antioxidants in AMD which warrant further study for application in clinical practice. Our current understanding is that natural antioxidants have the potential to improve or halt the progression of AMD, and tailoring therapeutics to the specific disease stages may be the key to preventing irreversible vision loss.

## 1. Introduction

Age-related macular degeneration (AMD) is a progressive disease affecting the macular region of the retina. Approximately 290 million individuals are predicted to be affected with AMD by 2040, with 110 million in Asia [1]. It was one of the major causes of blindness globally in 2020 [2] among individuals aged 50 and older, after cataract and glaucoma. The population-based longitudinal Beijing Eye Study revealed that visual impairment due to AMD was relatively uncommon in the adult Chinese population in rural and urban regions [3]. The Singapore Epidemiology of Eye Disease (SEED) Study reported that the prevalence of early-stage AMD in Singapore was similar to that of Australia, although Singaporeans had a milder spectrum of early AMD lesions (soft distinct drusen and noncentral location) compared to Australians [4]. According to a population-based cross-sectional study conducted by Cheung et al. [5], early-stage AMD is more common in Chinese and Indians than in Malays, whereas no apparent racial differences were observed in the prevalence of late-stage AMD.

AMD has no known cure at present. Disease progression is monitored using Amsler charts and scheduled clinic examination with the use of optical coherence tomography, which is a cross-sectional retinal scan. Treatment options such as intravitreal injection of antivascular endothelial growth factor (anti-VEGF), photodynamic laser therapy (PDT), or a combination of both, have been shown to improve vision and stabilize disease progression [6]. Nonetheless, concerns have been raised on potential ocular complications and systemic side effects of these treatments.

As oxidative damage, inflammation and neovascularization are key pathological events implicated in AMD, antioxidants such as active compounds, carotenoids, extracts and polysaccharides, flavonoids, formulations, vitamins and whole foods may have the potential to reverse or delay disease progression. The review aims to examine and synthesize the current research, i.e., published from 1 January 2011 to 31 October 2021, on the therapeutic roles of natural antioxidants in the treatment of AMD. We also discuss the reported molecular mechanisms of action of natural antioxidants, including their synergistic effects and efficacies, as well as protective mechanisms. This review provides a mechanistic framework for the role of antioxidants as therapeutics for AMD from preclinical studies to clinical trials.

### 1.1. Pathogenesis of AMD

AMD is a multifactorial disease. Several hypotheses have been proposed to explain the nature of AMD, such as aging, genetics and degeneration of photoreceptor-retinal pigment epithelium (RPE) complex. Many factors have been linked to an increased risk of developing AMD, including sex, ethnicity, iris pigmentation, hormones, hypermetropia, arthritis, type II diabetes, medications, body mass index, level of education, socioeconomic status, nutritional status, lifestyle (i.e., smoking and alcohol intake) and sunlight exposure [7]. Oxidative damage and inflammation are the key features shared by these events, as well as the driving forces in the pathogenesis of AMD. This is supported by Abokyi et al. [8], who highlighted the central role of oxidative damage in the retina that contributes to inflammation and angiogenesis.

Degeneration of retinal photoreceptors, retinal pigment epithelium, Bruch’s membrane (BrM) and choriocapillaris have been shown to be involved in the pathogenesis of AMD [9], culminating in the breakdown of the blood-retina-barrier and retinal degeneration. These changes occur in the macula and proceed through various stages (early, intermediate and advanced) over time.

AMD involves a variety of phenotypic changes in the posterior pole. There are two types of AMD, namely dry (nonexudative) AMD, resulting from atrophy of the retinal pigment epithelial layer, and wet (exudative) AMD, causing vision loss due to abnormal blood vessel growth [10]. The characteristic lesions are aggregations of lipid-containing extracellular particles in the RPE/BrM complex (drusen and basal deposits) that ultimately impact RPE and photoreceptor integrity. Drusens appear clinically as focal white-yellow deposits deep to the retina. They are divided into two main phenotypes, i.e., “hard” and “soft”, that are discernible by their edges and the level of risk they confer on progression of AMD [11].

Early-stage disease is characterized by soft or hard drusens, and/or irregular focal hypo- or hyper- pigmentation. Wet AMD is characterized by soft drusens in the early stages. Choroidal neovascularization (CNV) and subretinal fluid accumulation which eventually leads to scarring and loss of photoreceptors RPE cells occur in the advanced stages. In late stages of dry AMD, death of photoreceptors and RPE cells results in geographic atrophy (GA) over the macula. In both instances, the loss of photoreceptors in the macula, specifically, in the fovea region, leads to central vision loss.

#### 1.1.1. Features of Choroidal Neovascularization

**Figure 1:**
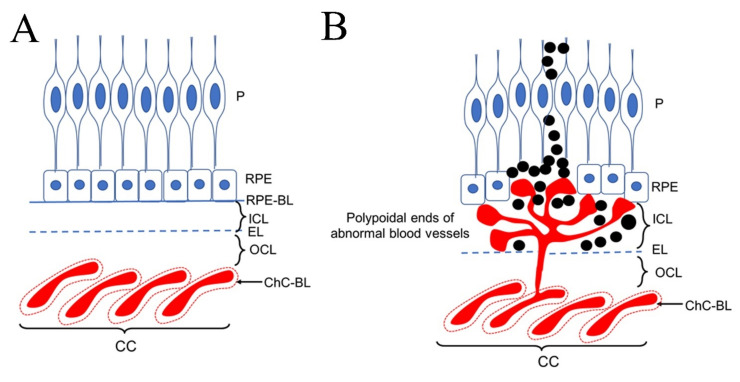
Schematic cross-sections of choriocapillaris (CC)-Bruch’s Membrane (BrM)-retinal pigment epithelium (RPE)-photoreceptor complex. (**A**) Normal eye. BrM, from the RPE to the choroid, consists of five distinctive layers: RPE basal lamina, inner collagenous layer, elastic layer, outer collagenous layer, and basement membrane of choriocapillaris. (**B**) Eye with AMD. Formation of new abnormal blood vessels by VEGF in the choroid and disturbance of integrity of BrM and RPE lead to subretinal fluid accumulation (indicated by black circles) and visual impairment in the late-stage AMD. Polypoidal choroidal neovascularization (CNV) in the form of small aneurysmal dilations of vessels resembling a cluster of grapes has a high risk of bleeding and leakage. P, photoreceptors; RPE, retinal pigment epithelium; RPE-BL, RPE basal lamina; ICL, inner collagenous layer; EL, elastic layer; OCL, outer collagenous layer; ChC-BL, choriocapillaris basal lamina.

**Figure 2:**
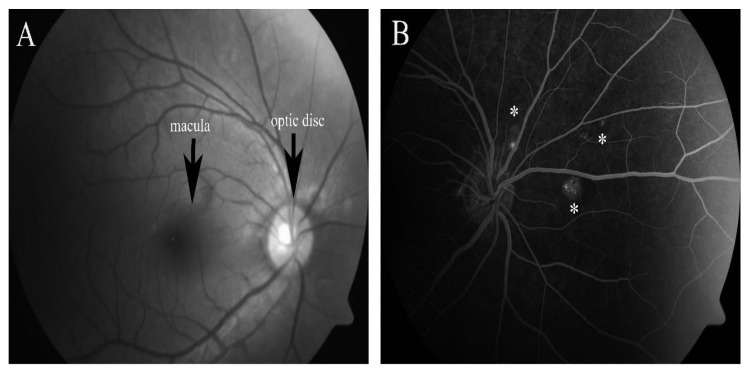
Representative fundus image: (**A**) Normal; (**B**) Wet age-related macular degeneration (AMD). Asterisk indicates drusen, which are lipid-containing aggregations found in the retinal pigment epithelium (RPE)/Bruch’s membrane (BrM) complex. (Original image from Kah-Hui Wong).

Choroidal neovascularization (CNV) is the manifestation of wet AMD and is defined by the growth of new blood vessels from the choroid that extend into the subretinal or sub-RPE space. Polypoidal choroidal vasculopathy (PCV), a subtype of CNV, is characterized by the presence of multiple vascular saccular dilations (polyps) in choroidal circulation [12]. As shown in Figure 1, abnormal vascularization causing exudation in eyes with PCV is consistently observed between an elevated RPE and BrM. The clinical manifestation includes multiple recurrent serosanguineous or hemorrhagic detachment of RPE and retina, usually around the optic nerve or in the central macula. CNV is one of the leading causes of irreversible severe loss of vision among the elderly. In younger adults (age 50 years and below), CNV can be idiopathic or secondary to underlying hereditary and acquired conditions, including angioid streaks, high myopia, and traumatic or inflammatory disorders. Assessments of CNV involve imaging the macula with optical coherence tomography (OCT), fundus fluorescein angiography (FFA) and indocyanine green angiography (ICGA). Hemorrhages, exudates, detachment of RPE or retina, and/or disciform scars are typical findings [13]. OCT is routinely used to assess the clinical response to anti-VEGF or PDT. Figure 2 shows two representative eyes with and without clinically detected CNV.

#### 1.1.2. Features of Geographic Atrophy

Geographic atrophy (GA) is an advanced form of dry AMD which is characterized by photoreceptor degeneration, leading to a loss of underlying RPE cells. The clinical manifestations include sharply delineated areas of severe depigmentation and the absence of RPE cells. Large choroidal vessels can be easily visualized on color fundus photography (CFP). Unlike CNV, GA usually spares the foveal center until late in its course [14].

### 1.2. Standard Treatment Options for Wet AMD

There are several treatment options for wet AMD, namely, photodynamic therapy (PDT), intravitreal injection of anti-VEGF (pegaptanib, ranibizumab, aflibercept and bevacizumab), and laser photocoagulation [15]. Photodynamic therapy was first introduced in the late 1990s [16] and involves the intravenous administration of Verteporfin (a photosensitizing agent approved by the US Food and Drug Administration (FDA) for ophthalmic use) at a dose of 6 mg/m^2^ of body surface area. Verteporfin is then activated with a monochromatic laser light (range, 689–691 nm) in the presence of oxygen, which shifts it to an electronically excited state [17]. This is followed by the generation of reactive oxygen radicals that cause local damage to the vascular endothelium to destroy new abnormal vessels. There have been major breakthroughs in the development of therapies targeting VEGF since the early 2000s. Intravitreal injection of anti-VEGF is performed using a 30-gauge needle at 3.5 to 4 mm posterior to the limbus under local anesthesia. The injection site is compressed by a cotton swab to avoid reflux [18]. The fundus is then examined to rule out any complications and to check the perfusion of the central retinal artery. The subsequent inhibition of VEGF blocks the formation of new abnormal vessels, resulting in vessels becoming porous, and in turn, ranibizumab has been shown to clinically improve visual acuity significantly [19,20]. The efficacy and safety of ranibizumab and PDT with verteporfin were evaluated in 423 patients with neovascular AMD in a phase III clinical trial: The Anti-VEGF Antibody for the Treatment of Predominantly Classic Choroidal Neovascularization in Age-Related Macular Degeneration (ANCHOR). The patients were randomly assigned to receive either 0.3 or 0.5 mg of ranibizumab on a monthly basis or active verteporfin therapy administered intravenously at 3-month intervals for 12 months. The proportion of patients who gained ≥15 letters from baseline to 12 months in the best corrected visual acuity (BCVA) test was 40.3%, 35.7%, and 5.6% in the 0.5 mg and 0.3 mg ranibizumab and verteporfin groups, respectively. The rates of serious adverse events after ranibizumab injections were reported to be low [19,21].

In addition, PDT in combination with anti-VEGF is considered a second-line treatment for patients who fail to respond to monotherapy with anti-VEGF; it works by stimulating polyp regression in PCV [22,23]. Combination therapy of ranibizumab and PDT [23,24] or bevacizumab and PDT [25,26] has been found to be more effective than monotherapy. In a meta-analysis of randomized controlled trials conducted by Wei et al. [27], there were no statistically significant differences in the parameters of BCVA, central retinal thickness (CRT), proportions of patients gaining ≥15 letters, incidences of ocular and systemic adverse events between patients who received a combination of bevacizumab and PDT, and patients who were treated with bevacizumab monotherapy. However, the need for monthly injections with bevacizumab was significantly lower in the combination therapy group compared to the monotherapy group. This is in line with the findings of Ito et al. [28] who reported that a combination of ranibizumab or aflibercept and PDT was effective in preserving or improving visual acuity and anatomical structures in patients with PCV evidenced by noticeable effect on choroidal thickness at a one-year follow-up.

On the other hand, the development of effective treatment options for dry AMD has not progressed to a similar extent. No effective treatment have yet been found to prevent the onset of GA and therefore dry AMD remains the largest unmet need in retinal disease management [29].

There is no effective cure for AMD and current therapeutic strategies focus only on symptomatic and supportive management, and limiting its progress [30]. The complexity in the neurochemistry of AMD suggests that there may be multiple therapeutic targets. Patients are usually reassured that progression of AMD is usually slow and they are likely to retain their independence even if reading vision is compromised. Other useful interventions may include smoking cessation, rehabilitation and low vision aids. Moreover, pharmacological breakthroughs have yet to be fully translated into clinical benefits for patients and medical practice, given the limited research on animal models.

### 1.3. Adverse Events following Standard Treatment for Wet AMD

Schnurrbusch et al. [31] evaluated the occurrence of complications following PDT with verteporfin for subfoveal CNV secondary to AMD and pathologic myopia in a retrospective case series. In general, PDT was well tolerated in patients with CNV secondary to AMD and in patients with pathologic myopia. However, moderate and transient adverse reactions were observed in some patients, including infusion-related back, chest or body pain, dyspnea and flushing during infusion, dyspnea alone, elevated blood pressure, and general pruritus. In addition, transient visual disturbances with haziness, blurriness, and flashing lights were experienced by 27.8% of patients. These complications lasted for about 3 days and were resolved spontaneously within days to weeks.

Tzekov et al. [32] demonstrated functional and morphological changes of the retina detectable for up to 9 months in a primate model of cynomolgus monkey following a single PDT treatment. The eye in this primate model is morphologically and functionally identical to the human eye, and has been shown to be responsive to PDT treatment [33]. Intermittent increase in subretinal fluid level, foveal thinning and loss of RPE can be present in the same area, causing clinical long-term effects in some patients. The incidence of damage to the neural retina overlaying the PDT-treated areas has been reported clinically [34,35,36]. Newman [37] revealed a higher incidence of adverse events with PDT when sealing the leakage site close to the fovea, including damage to the normal choriocapillaris and RPE leading to choroidal ischemia, RPE atrophy and secondary CNV.

Anti-VEGFs currently form the mainstay in the management of retinal diseases. Despite its promising efficacy in restoring vision, intravitreal injection of anti-VEGF is associated with devastating complications. Ophthalmologists should consider the potential ocular complications and systemic risks, while closely monitoring for adverse outcomes experienced by their patients throughout the administration of anti-VEGF [18]. The complications that are unrelated to underlying ocular disease include endophthalmitis, intraocular inflammation, rhegmatogenous retinal detachment, intraocular pressure elevation, ocular hemorrhage and systemic adverse events, whereas those that are related to underlying ocular disease (diabetic retinopathy, retinal vascular occlusions, AMD and other retinal diseases) include vitreoretinal fibrosis, development of tractional retinal detachment, central retinal artery occlusion, retinopathy of prematurity and development of secondary rhegmatogenous retinal detachment [38,39]. Furthermore, intravitreal injection of ranibizumab has been shown to have greater risk of developing cardiovascular disease [40,41]. In a study conducted by Singer et al. [42], the increase in complication rates is proportional to the number of injections required for two years or more.

Although anti-VEGFs have been found to stabilize vision in many patients, monotherapy was shown to be unable to achieve significant improvement in visual acuity in a substantial number of AMD patients through long-term management [43,44,45,46]. While it stabilized vision and reduced subretinal fluid and foveal thickness, anti-VEGF monotherapy was ineffective at reversing choroidal vascular changes and polypoidal lesions at 3-month follow-up [47], 9 to 18-month follow-up [48], and 12 to 30-month follow-up [49], which could lead to recurrence of exudative maculopathy.

### 1.4. Role of Natural Antioxidants for AMD

Accumulating scientific and clinical evidence reveals that chronic oxidative damage is one of the crucial factors in the pathogenesis of retinal degenerative diseases, including AMD [50,51]. Oxidative damage is an imbalance between pro- and antioxidant species, resulting in molecular and cellular damage. Excessive production of intracellular free radicals, namely the reactive oxygen species (ROS) and reactive nitrogen species (RNS) can react with and denature biological macromolecules (nucleic acids, lipids and proteins).

Substantial evidence from animal studies also indicated that prolonged light stimulation to the retina can cause accumulation of oxidative damage [52,53,54]. Exposure to ultraviolet (UV) radiation initiates oxidative DNA damage and inflammatory response in RPE. Taken together, these events cause overproduction and accumulation of lipofuscin and formation of toxic aggregates of amyloid-β (Aβ) peptides. Under physiological conditions, the cellular waste that includes lipofuscin, drusen and unnecessary proteins are eliminated in RPE through ubiquitin-proteasome system (UPS) and autophagy. Impaired autophagy in RPE may contribute to further accumulation of such aggregates. Such alterations are typically observed in drusen of AMD patients, denoting abnormal lipid- and protein-rich sub-RPE deposits [55]. Moreover, postmortem fundus examination and histopathology of samples from AMD patients revealed clinical signs of extensive free radical damage [50,56,57].

Several studies support the notion that nuclear factor erythroid 2-related factor 2 (Nrf2) play an active role in the regulation of autophagy. In response to oxidative damage, upregulation of Nrf2 signaling activates a complex antioxidant response which maintains cellular redox homeostasis. Understanding the regulatory mechanisms that control Nrf2 protein levels, along with the molecular mechanisms of UPS and autophagy, will guide future development of Nrf2-targeted therapeutics in AMD [8].

Consequently, antioxidant defense systems consisting of endogenous and exogenous antioxidants are required to combat oxidative damage for maintaining cellular redox homeostasis [58]. Exogenous antioxidants can be obtained from natural sources. Primary antioxidants include phenolic compounds, phenolic acids and their derivatives, flavonoids, tocopherols, phospholipids, amino acids, phytic acid, ascorbic acid, sterols and pigments. Phenolic compounds act as free radical terminators to upregulate the activity of endogenous antioxidant enzymes, and therefore indirectly attenuate oxidative damage [59,60,61]. Consistently, antioxidant-enriched diets have been shown to reduce the progression from dry to wet AMD [62].

## 2. Materials and Methods

### 2.1. Search Strategy

A literature search of the electronic databases PubMed, Web of Science and Google Scholar for peer-reviewed articles published between 1 January 2011 and 31 October 2021 was undertaken. The following search terms were used: (“advanced neovascular age-related macular degeneration” OR “age-related macular degeneration” OR “dry macular degeneration” OR “neovascular age-related macular degeneration” OR “non-neovascular age-related macular degeneration” OR “wet macular degeneration”) AND (alga OR algae OR basidiomycetes OR “Chinese herb” OR “complementary and alternative medicine” OR decoction OR fungi OR herb OR “herbal product” OR “herb remedies” OR mushroom OR “natural antioxidant” OR “natural product” OR plant OR shrub OR “traditional Chinese medicine”). The structured search strategy was used to identify all articles that evaluated the therapeutic roles of natural antioxidants in AMD.

### 2.2. Eligibility Criteria

Studies were considered eligible if they met the following inclusion criteria: (i) preclinical (in vitro and in vivo studies) and clinical studies, (ii) study model of AMD as the primary disorder, and (iii) articles published in English. The exclusion criteria included: (i) in silico studies, (ii) in ovo studies, (iii) synthetic antioxidant, (iv) review articles, (v) meta-analysis, (vi) conference abstracts or proceedings, and (vii) articles written in languages other than English.

### 2.3. Data Extraction and Analysis

After removing duplicates, titles and abstracts were screened based on the eligibility criteria. Disagreements on the eligibility of the study or on the extraction of data were resolved through discussions. The findings were extracted independently and narrated to the best of our ability considering the inconsistencies in the methodology or experimental designs of the retrieved studies.

## 3. Results

### Study Selection

The literature search yielded 380 publications from PubMed, Web of Science and Google Scholar. After removing duplicate studies, 272 studies remained and were further screened by titles and abstracts. Overall, 163 articles were retrieved for further assessment and evaluation, of which 63 were excluded according to the exclusion criteria regarding study design and language. A total of 100 eligible studies (82 preclinical studies and 18 clinical studies) were included in this review.

## 4. Discussion

Most of the preclinical studies were based on the adult human retinal pigment epithelial cell line-19 (ARPE-19) and light-induced retinal degeneration models of oxidative damage. Although ARPE-19 cells lack melanin, the model is widely used to study the cell biology, pathological conditions and pharmacology of the retina. The acute model of light-induced retinal degeneration employs short exposure durations (seconds to minutes) to bright white light culminating in photoreceptor apoptosis and vision loss. Here, we review the randomized controlled trials investigating the efficacy of dietary supplements in patients with various stages of AMD. A randomized study design is often viewed as the gold standard for determining the true relative efficacy of an intervention, as it can eliminate the influence of unknown or immeasurable confounding variables, which can lead to biased estimation of the treatment effect.

### 4.1. Active Compounds

The chemical structures, findings and mode of action of active compounds in preclinical and clinical models are summarized in Table 1.

#### 4.1.1. Alkaloids

Plants are regarded as the oldest source of alkaloids, including morphine, quinine, strychnine and cocaine. Allicin is an organosulfur compound present in garlic, whereas berberine is an isoquinoline alkaloid present in the roots, rhizomes and stems of *Coptis chinensis.* Chopping or crushing garlic releases allicin, which has a pungent smell. Preclinical evidence has revealed the modulation of ROS-associated enzymes involving superoxide dismutase (SOD), NADPH oxidase 4 (NOX4), NAD(P)H dehydrogenase quinone 1 (NQO1), Nrf2; [63] caspase-3/7, adenosine monophosphate-activated protein kinase (AMPK)-mediated autophagy; [66,67] RHO, retinal pigment epithelium-specific 65 (RPE65), monocarboxylate transporter 3 (MCT3) as well as inflammatory markers (heme oxygenase-1 (HMOX1), ceruloplasmin (CP), catalase (CAT), glutathione peroxidase-1 (GPx-1), SOD2 and allograft inflammatory factor 1 (AIF1) [68] contributing to the protective effects of allicin and berberine.

#### 4.1.2. Curcumin

Curcumin is a lipophilic polyphenol present in *Curcuma longa* (turmeric) roots. Turmeric has been gaining tremendous attention due to its antioxidant abilities by scavenging ROS, including superoxide radicals (O_2_^•^), hydrogen peroxide (H_2_O_2_), hydroxyl radicals (OH^•^) and singlet oxygen (^1^O_2_). The positive outcomes of its therapeutic effects in cataract and diabetic retinopathy are well documented [88].

Curcuminoids are yellow pigments which confer the characteristic color to the rhizome. Curcuminoids are linear, diarylheptanoid molecules including curcumin and related compounds [89]. Other major constituents include ar-turmerone, turmerone, curlone, Zingiberene and Curcumene. There is ample evidence from in vitro tests based upon the human ARPE-19 cell line [71,73,74], as well as induced pluripotent stem cells (iPSCs)-derived RPE cells [90], to demonstrate the protective effects of curcumin against the development of AMD. Muangnoi et al. [74] pioneered the investigation on a succinate ester prodrug of curcumin, designated as curcumin diethyl disuccinate against oxidative damage induced in human ARPE-19 cells. Modulation of multiple molecular targets has been found to be associated with its protective effects, namely the p44/42 (extracellular-signal-regulated kinase (ERK)), Bcl2 associated X (Bax), B-cell lymphoma 2 (Bcl-2), heme oxygenase-1 (HO-1) and NQO1. This discovery may make it possible to overcome challenges related to the poor solubility of curcumin resulting in low bioavailability.

A recent finding by Allegrini et al. [91] highlighted an emerging strategy for the treatment of neovascular AMD. A curcumin supplement consisting of 95% curcuminoids, Age-Related Eye Disease Study 2 (AREDS2) components, astaxanthin and resveratrol, in combination with intravitreal injection of anti-VEGF, was shown to improve functional outcomes in a retrospective case-control study. Curcuma, used in an adjuvant setting, would be necessary to reduce the need for ongoing injection therapy. However, recall bias occurs most often in case-control studies. These studies may prove an association, but they do not demonstrate causation; this can be overcome by cohort studies. Despite promising findings, further evidence is needed to evaluate the impact of curcuma in human clinical trials of AMD. A lack of clinical trials evaluating the safety and efficacy of the adjuvant setting can be a point of concern for evidence-based research in alternative medicine.

#### 4.1.3. Ginsenoside

*Panax ginseng*, or Korean ginseng, has been traditionally used for several millennia in East Asian countries. Ginseng refers to the root of *P. ginseng.* Ginsenosides are the major pharmacologically active ingredients of ginseng. Approximately 40 structurally divergent ginsenosides have been isolated and identified from ginseng.

Betts et al. [79] revealed the efficacy of ginsenoside-Rb1 in triggering cell proliferation and reducing the secretion of VEGF in human ARPE-19 cells. This could be mediated by estrogen receptor signaling mechanisms based on a report indicating that high levels of estrogen decreases extracellular level of VEGF in the retinal capillary cells of rhesus monkey. The observation could further support the clinical application of ginsenoside-Rb1 in reducing the frequency of anti-VEGF injections. On the other hand, Lee et al. [80] demonstrated an improvement of hydraulic and diffusional transport across BrM by ginsenosides. BrM and RPE form a serially coupled transportation system for nutrients and waste products. Aging causes gross anatomical changes in these compartments, and further exaggerated in AMD. Therefore, direct targeting of transport systems serves as a therapeutic intervention in delaying the progression of AMD. However, the findings have not been validated in clinical trials.

#### 4.1.4. Other Active Compounds

Numerous compounds, namely, artemisinin (a lactone isolated from *Artemisia annua*) [64], astragaloside (a cycloartane-type glycoside isolated from *Astragalus membranaceus*) [65], celastrol (a quinone methide triterpene) [70], carnosic acid (a phenolic diterpene isolated from rosemary extract) [69], diarylheptanoids isolated from *Curcuma comosa* [75], diphlorethohydroxycarmalol (a phlorotannin isolated from brown macroalga *Ishige okamurae*) [76], FLZ (a novel synthetic cyclic analogue of natural squamosamide isolated from *Annona glabra*) [77,78], glycyrrhizin (a glycoside isolated from licorice roots (*Glycyrrhiza* glabra)) [81], GPETAFLR (a biopeptide isolated from *Lupinus angustifolius*) [82], gypenosides (dammarane-type triterpene saponins isolated from *Gynostemma pentaphyllum*) [83], kinsenoside isolated from *Anoectochilus roxburghii* [84], phillyrin (a lignan isolated from dried fruit of *Forsythia suspense*) [85], rosmarinic acid isolated from *Rosmarinus officinalis* [86] and total saponins isolated from rhizomes of *Paris polyphylla* [87] also demonstrated promising protective effects against oxidative damage in preclinical models.

The molecular signaling mechanisms governing these effects were largely related to AMP-activated protein kinase (artemisinin),TNF receptor-associated factors 5 (TRAF5) (astragaloside), nuclear factor kappa-light-chain-enhancer of activated B cells (NF-Κb) (astragaloside, kinsenoside and FLZ), intracellular 70-kDa heat shock proteins (HSP70) (celastrol), modulation of inflammatory cytokine (celastrol and GPETAFLR), antioxidant response elements including Nrf2 (carnosic acid, diarylheptanoids, glycyrrhizin, phillyrin and total saponins), modulation of DNA damage marker, pro-apoptotic and anti-apoptotic protein (diphlorethohydroxycarmalol, glycyrrhizin, phillyrin and total saponins), protein kinase B (Akt) and nuclear factor of kappa light polypeptide gene enhancer in B-cells inhibitor, alpha (IκBα) (FLZ).

In addition, Biswas et al. [83] revealed that gypenosides may have potential for the treatment of patients with early-stage AMD by enhancing the ability of efflux pathways to remove cellular cholesterol from RPE cells. Apolipoprotein E (APOE) and ATP binding cassette subfamily A member 1 (ABCA1), cholesterol efflux genes and cholesterol accumulation beneath the RPE cells have been shown to contribute to the pathogenesis of AMD [92]. However, definitive proof remains elusive on how cholesterol efflux influences the accumulation of lipids in sub-RPE deposits.

A recent, remarkable trial on New Zealand white rabbits demonstrated the efficacy of therapeutically designed rosmarinic acid-poly lactic-co-glycolic acid (PLGA) implants in impeding ocular neovascularization. This strategy takes a long-term view toward the possible use of PLGA, a biodegradable polymer as an implantable intravitreal device to promote prolonged and controlled release of rosmarinic acid in vitreous humor [86].

### 4.2. Carotenoids

The chemical structures, findings and mode of action of carotenoids in preclinical and clinical models are summarized in Table 2.

Carotenoids are found as fat soluble and colored-pigments in yellow-orange fruits and vegetables, as well as in some varieties of dark green vegetables. Approximately 750 structurally different carotenoids have been isolated from natural sources to date. Seven types are commonly found in the human diet and plasma serum, namely, lutein, zeaxanthin, α-carotene, β-carotene, lycopene, *meso*-zeaxanthin and cryptoxanthin [99]. The lipid-soluble pigments promote stabilization function with respect to the plasma membrane and modification of diffusion barrier to and across the membrane. The conjugated polyene chromophore determines not only the light absorption properties, but also the photochemical properties of the molecule and consequent light-harvesting and photoprotective action. The polyene chain is responsible for the chemical reactivity of carotenoids toward blue light absorption, quenching of excited singlet and triplet states by molecular oxygen and free radical scavenging, and hence for any antioxidant role.

Lutein and zeaxanthin have been demonstrated to be protective against the development of AMD in numerous studies. With respect to key molecules in signaling pathways, inhibition of phosphorylation of p38 mitogen-activated protein kinase (p38 MAPK) and c-Jun N-terminal kinase (JNK)1/2 by lutein and zeaxanthin has been found to play a protective role in a cellular model of UVB irradiation-induced oxidative damage [95]. In a recent study by Orhan et al. [93] using an animal model of light-induced retinal degeneration, β-cryptoxanthin was shown to attenuate oxidative damage to mitochondria and inflammation through the inhibition of inflammatory cytokine, modulation of apoptotic pathways and mitochondrial stress markers. Several studies have been performed to investigate the association between α-carotene, β-carotene, and lycopene; and risk of AMD. However, their results were inconsistent [100,101].

Carotenoids are concentrated in the macula or central region of the retina, also known as macular pigment [98]. The yellow coloration of the macula lutea is attributed to the presence of macular pigment in the photoreceptors [102]. Cho et al. [103] reported an inverse association of lutein/zeaxanthin with advanced AMD. These results are in accordance with clinical trials conducted by Age-Related Eye Disease Study 2 (AREDS2) Research Group (2013) [104,105]. Nevertheless, observational data from AREDS2 did not achieve sufficient evidence to demonstrate the beneficial effects of lutein and zeaxanthin when added to the original Age-Related Eye Disease Study (AREDS) formulation in reducing the risk of progression to advanced AMD.

### 4.3. Extracts and Polysaccharides

The chemical structure, findings and mode of action of extracts and polysaccharides in preclinical models are summarized in Table 3.

Several preclinical models have been used to explore the potential role of aqueous and ethanol extracts of medicinal plants in the mitigation of oxidative damage associated with AMD. In each of these models, early indicators include pigmentary and structural RPE changes, as well as retinal and choroidal thinning, contributing to the pathogenesis of AMD. Ethanol extracts of *Arctium lappa* or burdock [106], bilberry [108], *Bucida buceras* [109], *Centella asiatica* or pennywort [110], *Diospyros kaki* or persimmon [112], lingonberry or cowberry [108], *Melissa officinalis* or lemon balm [125], *Pueraria lobate* [126], *Solanum melongena* or eggplant [130], *Tribulus terrestris* or puncture vine [131], and aqueous extracts of *Vaccinium uliginosum* or bog bilberry [132], purified *Emblica officinalis* or amla extract [113], grape skin extract [119], Saudi *Origanum vulgare* extract-mediated gold nanoparticles [129] and *Garcinia cambogia* or Malabar tamarind extract [118] have been revealed to prevent the oxidative damage involved in the development of AMD. These findings have generated interest in botanical substances with antioxidant capabilities associated with the upregulation of antioxidant enzymes, downregulation of hypoxia response element (HRE) sequence and modulation of endoplasmic reticulum (ER) stress and unfolded protein response. Importantly, activation of transcription factor NF-κB and inflammatory cytokine tumor necrosis factor alpha (TNF-α) has been linked to apoptosis, which could provide insight into the critical effector pathways regulating the therapeutic intervention in AMD. Interestingly, these pathways often intersect with the mechanism of action of numerous botanical substances, namely asiaticoside [110], carotenes, triterpenes, steroids, lactonic groups [109], flavonoids including chlorogenic acid and proanthocyanidin [108,130,132], hydroxycinnamic acid derivatives [125], hydroxycitric acid [118], phenolic acids including *trans*-resveratrol [107], tannins, amines, amino acid and saponins [109].

Wang et al. [129] demonstrated the remarkable protective effects of synthesized gold nanoparticles of Saudi *O. vulgare* extract in the prevention of early-stage or dry AMD by inhibiting angiogenesis and apoptosis, and increasing the expression of pro-inflammatory cytokines. Nanotechnology has the potential to make a significant impact on pharmacological and surgical interventions. Nanocarriers are designed to overcome the difficulties associated with anatomical and physiological barriers limiting the access to retina.

Mounting evidence suggests that overexposure to blue light induces a significant increase in ROS production, contributing to the loss of photoreceptors, lipid peroxidation and cell apoptosis. As a major component of drusen, N-retinylidene-N-retinylethanolamine (A2E) is a metabolic by-product of RPE cells and a blue light absorbing retinal chromophore that accumulates with age. The interaction of blue light, A2E and photoreversal of bleaching will further aggravate photochemical damage and cause the activation of inflammatory reactions, DNA damage and inhibition of mitochondrial and lysosomal function. Interestingly, ethanol extract of *A. lappa* [106], *C. asiatica* [110], and *S. melongena* [130], aqueous extract of *V.* *uliginosum* [132,133] and grape skin extract [119] displayed cytoprotective effects against A2E oxidation induced by blue light in ARPE-19 cells.

Various lines of evidence have also revealed a downregulation of expression of VEGF family members at mRNA and protein levels in halting the development of pathological angiogenesis in CNV. Bilberry anthocyanin-rich aqueous extract [107], *G. cambogia* extract [118], purified *E. officinalis* [113], lactoferrin [120], red wine extract [127], and Saudi *O. vulgare* extract-mediated gold nanoparticles [130] have been shown to downregulate VEGF in preclinical models. VEGFA is the most potent mediator of both retinal and choroidal angiogenesis, and its inhibition through the regulation of hypoxia-inducible factor (HIF)/VEGF axis indicates the role of HIF in maintaining cellular homeostasis in response to changes in the oxygen status. HIF-1α is a major regulator of angiogenesis and is expressed ubiquitously to control various genes such as VEGF, BCL2 interacting protein 3 *(*BNIP3) and phosphoinositide-dependent kinase 1 (PDK1).

Fucoidan is a sulfated polysaccharide of long-branched chains of sugars with high fucose content extracted from brown macroalgae. Fucoidans have recently been used for the treatment of wet AMD in various in vitro models studying VEGF expression. Crude fucoidan of *Fucus distichus* subsp. *evanescens* [111]; fucoidans of *Fucus vesiculosus* [114], *Fucus vesiculosus*, *F. distichus* subsp. *evanescens, Fucus serratus, Laminaria digitata, Saccharina latissimi* [115,117] and *Laminaria hyperborean* [116] have been shown to reduce VEGF expression. Intriguingly, Dithmer et al. [114] revealed a novel antiangiogenic approach of fucoidans in preventing the secretion of VEGF in ARPE-19 cells, primary porcine RPE cells and RPE/choroid perfusion organ culture incubated with bevacizumab. Therefore, fucoidans can be developed as VEGF antagonists in the treatment of angiogenesis-dependent diseases as in CNV. However, the molecular mechanisms mediating the regulation of VEGF has not yet been entirely elucidated.

*Lycium barbarum* (wolfberry, goji berry) has been used for more than 2000 years in traditional Chinese medicine (TCM). Its medicinal values are documented in the Pharmacopoeia of the People’s Republic of China. Indeed, the polysaccharides which comprise 5–8% of dried fruits have been reported to possess antioxidant, anti-inflammatory and anti-apoptotic effects. Accumulating evidence suggests that *L. barbarum* may enhance macular health and prevent AMD [134]. The polysaccharides of *L. barbarum* have revealed potent preclinical efficacy against AMD [122,123,124]. Modulation of pro-apoptotic genes (Bax and Bcl-2), upregulation of antioxidant genes (Nrf2 and thioredoxin reductase 1 (TrxR1)), downregulation of DNA repair gene [poly (ADP-ribose) polymerase family member 14 (PARP14)] and restoration of antipyroptosis activity contributed to the protective effect of polysaccharides against photoreceptor apoptosis in preclinical models.

### 4.4. Flavonoids

The chemical structures, findings and mode of action of flavonoids in preclinical models are summarized in Table 4.

Anthocyanins are the water-soluble pigments responsible for the red, blue and purple colors observed in plants, flowers and fruits. Chromophores and glycosides anthocyanins, namely, pelargonidin, cyanidin, delphinidin, peonidin, petunidin and malvidin, are the most common anthocyanidins distributed in plants. Anthocyanin extract from blueberry, malvidin, malvidin-3-glucoside and malvidin-3-galactoside have been found to increase the level of endogenous antioxidant enzymes while decreasing ROS and malondialdehyde (MDA) formed during oxidative degeneration through the activation of Akt and inhibition of MAPKs in an in vitro model of oxidative damage [135]. Silvan et al. [95] also demonstrated the protective effects of cyanidin-3-O-glucoside through the inhibition of MAPKs (JNK1/2 and p38 MAPK) phosphorylation. These observations support the hypothesis that dietary supplements rich in anthocyanins may prevent AMD progression through antioxidant mechanisms.

Oxidative damage-induced inflammation leading to photoreceptor apoptosis and retinal degeneration can be reversed by quercetin. The associated molecular mechanisms and signaling pathways are well-elucidated in preclinical models. Inhibition of inflammatory cytokines and mediators [136,142,143], regulation of antioxidant enzymes [136,142] and inhibition of c-Jun/c-Fos heterodimerization regulated by activator protein-1 (AP-1) [144] have been shown to play important roles in the progression of AMD. AP-1 is a dimeric complex that is composed of heterodimers of the Fos and Jun family of proteins. Activation of AP-1, particularly the c-Jun transcription factor has been implicated in the induction of apoptosis activated by extracellular stimuli, whereas c-Fos triggers light-induced photoreceptor apoptosis.

Resveratrol (3,5′,4-trihydroxystilbene) is a polyphenol phytoalexin found in citrus fruits. Under various models of oxidative damage using human RPE cells, resveratrol has been demonstrated to be protective against the development of AMD by regulating the gene expression of antioxidant enzymes [147], Notch 4 signal transduction [145], vimentin [147] and MAPK expression [145,148]. Notch signaling is essential for vascular morphogenesis in response to ischemia by laying down primary vascular plexus for arterial specification [149]. Activation of MAPKs are implicated in RPE cell homeostasis following oxidative damage [150,151]. Moreover, resveratrol has been shown to inhibit the proliferation, migration and network formation of activated choroidal endothelial cells through the activation of p53 and inactivation of Akt in choroidal endothelial cells, leading to its antiproliferative and antimigratory properties [146].

Furthermore, multiple pro- and anti- apoptotic proteins were found to be regulated in the retina of a light-induced retinal degeneration model, indicating the therapeutic potential of resveratrol supplementation in preventing retinal degeneration caused by exposure to direct sunlight and several artificial light sources [146]. Intriguingly, resveratrol has been shown to inhibit bevacizumab expression and secretion in ARPE-19 cells incubated with resveratrol and bevacizumab [145]. The findings pave the way for future research design to explore the synergistic effects of resveratrol and anti-VEGF for patients with neovascular AMD in an anticipation to minimize the complications of anti-VEGF treatment regime.

Other flavonoids, namely, chlorogenic acid, epigallocatechin gallate, fisetin, homoisoflavonoids, kaempferol, luteolin and proanthocyanidins, also demonstrated promising protective effects against oxidative damage in preclinical models through the downregulation of matrix metalloproteinase (MMP)-9 and VEGF [137], inhibition of transcription factor NF-κB activation [136], inhibition of inflammatory cytokines [138] and modulation of apoptotic pathways [140].

### 4.5. Formulations

The findings and mode of action of the formulations in preclinical and clinical models are summarized in Table 5.

The Age-Related Eye Disease Study Research Group (ARED) [168] standardized a clinical protocol in 2000, giving rise to a formulation catering to patients with moderate- to advanced-stage AMD. The aim of this prospective multicenter nonrandomized study was to re-appraise the concept of antioxidants in the prevention and management of AMD. Subsequently, the original formulation was re-examined and completed in 2012 as AREDS2. The goal was to evaluate the effect of adding omega-3 fatty acids, replacing beta-carotene with lutein-zeaxanthin, and eliminating or lowering the zinc in AREDS. AREDS2 demonstrated a 10% reduction in the progression of intermediate dry AMD to advanced forms of atrophic and neovascular AMD compared to placebo [104].

Wong et al. [152] presented the combined effects of AREDS and nontraditional antioxidants (rosemary or its active compounds, carnosic acid or ursolic acid) in delaying the progression of AMD in an animal model of light-induced retinal degeneration. Chronic administration of the combination may be a useful adjunct to the therapeutic benefit of AREDS in slowing the loss of photoreceptor cells and the progression of AMD to advanced disease by regulating retinal gene expression, and increasing rhodopsin, rod S-antigen, cone opsin and cone arrestin.

As macular pigment consists of constituents derived solely from the diet, dietary modification has been postulated to promote potential progressive improvement in macular pigment optical density [169]. Consistent with the theory, the clinical efficacy of milk-based formulation designated as Lacto-Wolfberry [157] and whole fruit of *L. barbarum* [170] have been evaluated in randomized double-blinded placebo-controlled involving healthy elderly subjects and patients with neovascular AMD, respectively. The 90-day supplementation showed a striking ability to sustain visual function and delaying macular degeneration associated with soft drusen and areas of hypopigmentation. Recently, RESVEGA^®^ has been revealed to promote disruption of antiangiogenic action targeting on VEGF and its receptor, vascular endothelial growth factor receptor 2 (VEGFR-2) [162]. The formulation contains *trans*-resveratrol and omega-3 fatty acids, among other nutrients. The observation highlights the importance of AP-1 transcription factors in the regulation of VEGF and VEGFR-2 levels, and disrupting the dissociation of VEGF-R2/caveolin-1(CAV-1) complex into lipid rafts following VEGF stimulation for the therapeutic efficacy.

Further, the depletion of omega-3 fatty acids has been suggested as one of the causes of oxidative damage to the retina, leading to photoreceptor degeneration and accumulation of drusen in the sub-RPE or subretinal space. Omega-3 fatty acids are found in plants and marine-based foods in short- (alpha-linolenic acid [ALA]) and long- chain (eicosapentaenoic acid [EPA], and docosahexaenoic acid [DHA]) forms. In two cohort studies, omega-3 fatty acids incorporated in the regular diet has been revealed to reduce the risk and confer protection against various stages of AMD. The study population was derived from the Blue Mountains Eye Study and National Academy of Sciences-National Research Council World War II Veteran Twin Registry. Evaluation was performed by grading of retinal photographs and visual acuity test [171,172]. In an open-label pilot study in patients with dry AMD, a liquid formulation of omega-3 concentrate has been reported to enhance visual function following 6 weeks of supplementation [155]. Nevertheless, there was no evidence from randomized trials to justify the effectiveness of the interventions.

In addition, the synergistic therapeutic effects of TCM are mainly derived from the complex interactions between multiple active constituents within the herbal formulations. As such, Mingjing granule that consists of *Radix astragali*, *Salvia miltiorrhiza*, *Fructus lycii, Ecliptae herba,* Pollen Typhae and Cirsii Japonici Herba Carbonisata [158], and ZQMT, a traditional Chinese Patent Medicine consists of *Rheum officinale*, *Panax notoginseng, S. miltiorrhiza, Eclipta prostrata, Ilex pubescens, Rehmannia glutinosa, Radix Paeoniae Rubra*, *Paeonia suffruticosa, Scutellaria baicalensis, Fructus ligustri lucidi* and Leonuri Semen [166] have been demonstrated to reduce the frequency of intravitreal injection of ranibizumab in patients with neovascular AMD. Likewise, combination of Fufang Xueshuantong with ranibizumab reduced the thickness of RPE–Bruch’s membrane complex thickness in neovascular AMD [154]. The preparation consists of *P. notoginseng*, *Radix astragali*, *S. miltiorrhiza* and *Radix Scrophulariaceae.*

On the other hand, Du et al. [153] recently developed a novel ophthalmic preparation known as Chuanqi ophthalmic microemulsion in situ gel. Topical administration of the preparation, which consists of *Ligusticum chuanxiong* and *A. membranaceus* var. *mongholicus*, can be transported from conjunctival/cornea surface to the retina by three different routes; the transvitreal, uvea-scleral and periocular routes in an animal model of dry AMD induced by sodium iodide. Additionally, a patented pharmaceutical composition (Patent No: WO2012079419) demonstrated a positive impact on the course of CNV based on animal and human trials. Its main ingredients include *A. membranaceus*, *Angelica sinensis*, *Poria cocos*, *Fritillaria thunbergii, Panax pseudoginseng,* charred Radix et Rhizoma Rhei, Pollen Typhae and *Curcuma aromatic* [161]. Shihu Yeguang, a formulation consisting of 24 herbs has been revealed to prevent the development of bright light-induced photoreceptor degeneration by suppressing photo-oxidative stress-induced apoptosis [164]. This approach greatly bridges the gap between TCM and modern medicine by encouraging further studies into the synergistic actions of TCM.

Other formulations that offer protection against the development of macular degeneration are lutein formulation that contains *Calendula officinalis, Lycium barbarum, Vaccinium myrtillus, Cassia obtusifolia,* and *Rhodiola rosea* [156], nanomicellar drop formulation of curcumin [159], ophthalmic drop formulation that contains nanoparticles of diosgenin extracted from the tubers of *Dioscorea* wild yam [160], resveratrol formulation [163] and Triphala, a polyherbal Ayurvedic medicine consists of dried fruit powder of *Terminalia chebula*, *Terminalia bellerica* and *Phyllanthus emblica* [165].

### 4.6. Vitamins

The chemical structure, findings and mode of action of vitamin B6 in preclinical models are summarized in Table 6. Early clinical and epidemiological studies predicted associations between vitamins and AMD [100,173]. A diet rich in multivitamins may prevent or delay progression to advanced-stage AMD, particularly the CNV. In addition, the effects of vitamin B6 was recently verified in preclinical models of oxidative damage [174]. As a component of rice bran, vitamin B6 was shown to suppress retinal neovascularization through the inhibition of HIF and decreased mRNA expression of VEGF. HIF promotes an adaptive transcriptional response to hypoxia, and as such, is a major regulator of immune cell survival and function. HIF is an oxygen-sensitive dimeric transcription factor that promotes an adaptive response to hypoxia by regulating essential inflammatory functions of immune cells.

### 4.7. Whole Foods

The findings and mode of action of whole foods in preclinical and clinical models are summarized in Table 7.

A2E, N-retinyl-N-retinylidene ethanolamine; ABCA1, ATP binding cassette subfamily A member 1; ABCG1, ATP binding cassette subfamily G member 1; Aif1, allograft inflammatory factor 1; AIFL, apoptosis-inducing factor-like; Akt, protein kinase B; AMD, age-related macular degeneration; AMPK, adenosine monophosphate-activated protein kinase; ANG, angiogenin; ANGPTL3, angiopoietin-like protein 3; AP-1, activator protein-1; APH1A, Aph-1 homolog A, gamma-secretase subunit; APH1B, Aph-1 homolog B, gamma-secretase subunit; APPBP2, amyloid protein-binding protein 2; ARE, antioxidant response element; AREDS, Age-Related Eye Disease Study; AREG, amphiregulin; ARPE-19, human retinal pigment epithelial cell line-19; ATF, activating transcription factor; BAP, biological antioxidant potential; Bax, Bcl2 associated X; Bcl-2, B-cell lymphoma 2; BCVA, best corrected visual acuity; BiP, binding immunoglobulin protein; Bnip3, BCL2 interacting protein 3; c-Abl, tyrosine-protein kinase ABL; CAT, catalase; Cav-1, caveolin-1; CCL2, C–C motif chemokine ligand 2; CCNA2, cyclin A2; CD31, cluster of differentiation 31; CDC42, cell division control protein 42 homolog; CDK2, cyclin-dependent kinase 2; CEP, carboxyethylpyrrole; cFXST, Fufang xueshuantong; CHOP, C/EBP homologous protein; CNV, choroidal neovascularization; CoCl_2_, cobalt (II) chloride; COX-2, cyclooxygenase 2; CP, ceruloplasmin; CREB, cAMP response element-binding protein; CX3CR1, C-X3-C motif chemokine receptor 1; CXCL8, C-X-C motif chemokine ligand 8; CYP27A1, cytochrome P450 family 27 subfamily A member 1; CYP46A1, cytochrome P450 family 46 subfamily A member 1; d-ROM, diacron-reactive oxygen metabolites; DHA, docosahexaenoic acid; DLL4, delta-like 4; EGFR, epidermal growth factor receptor; EGR1, growth response 1; EIF2α, eukaryotic initiation factor-2α; EPA, eicosapentaenoic acid; ERG, electroretinogram; ERK, extracellular-signal-regulated kinase; GCLC, glutamate cysteine ligase catalytic subunit; GCLM, glutamyl cysteine ligase modifier subunit; GFAP, glial fibrillary acidic protein; GNG11, G protein subunit gamma 11; GPx, glutathione peroxidase; GRP, glucose regulatory protein; GSDMD, gasdermin D; GSH, glutathione; GST-pi, glutathione S-transferase pi; GSSG, oxidized glutathione; HIF, hypoxia-inducible factor; HNE, 4-hydroxynonenal; HO-1 or HMOX1, heme oxygenase-1; HRECS, human retinal microvascular endothelial cells; HUVECs, human umbilical vein endothelial cells; Iba1, ionized calcium binding adaptor molecule 1; ICAM-1, intercellular adhesion molecule-1; IFNγ, interferon gamma; IGFBP, insulin-like growth factor binding protein; IκBα, nuclear factor of kappa light polypeptide gene enhancer in B-cells inhibitor, alpha; IL, interleukin; INOS, inducible nitric oxide synthase; IP, intraperitoneal; IV, intravitreal injection; JNK, c-Jun N-terminal kinase; Keap1, Kelch-like ECH-associated protein 1; LC3, microtubule-associated protein light chain 3; LDLR, low density lipoprotein receptor; LPO, lipid hydroperoxide; LPS, lipopolysaccharides; LXR-α, liver X receptor alpha; MAPK, mitogen-activated protein kinase; MCP-1, monocyte chemoattractant protein-1; MCT3, monocarboxylate transporter 3; MDA, malondialdehyde; MEK, mitogen-activated protein kinase kinase; MIP-1β, macrophage inflammatory protein-1β; MMP, matrix metalloproteinase; MNU, N-Methyl-N-nitrosourea; MT-RNR2, mitochondrially encoded 16S RRNA; NCAM, neural cell adhesion molecule 1; NCSTN, nicastrin; NE, not evaluated; NF-κB, nuclear factor kappa-light-chain-enhancer of activated B cells; NFKBIA, NFKB inhibitor alpha; NLRP3, NLR family pyrin domain containing 3; NOX4, NADPH oxidase 4; NQO1, NAD(P)H dehydrogenase quinone 1; Nrf2, nuclear factor erythroid 2-related factor 2; Ocln, occludin; Olr425, olfactory receptor 425; OMM-1, uveal melanoma cell line; OPRK, opioid receptor kappa; PAI-1, plasminogen activator inhibitor 1; PARP1, poly(ADP-ribose) polymerase 1; PARP14, poly(ADP-ribose) polymerase 14; PDGF, platelet-derived growth factor; PDK1, pyruvate dehydrogenase kinase 1; PECAM, platelet endothelial cell adhesion molecule; PGC-1α, peroxisome proliferator-activated receptor gamma coactivator 1-alpha; PO, per oral; POLD1, DNA polymerase delta 1; PPAR, peroxisome proliferator-activated receptor; Prx2, peroxiredoxins 2; RANTES, regulated upon activation, normal T cell expressed and presumably secreted; RELA, v-rel avian reticuloendotheliosis viral oncogene homolog A; RGD1564999, isopentenyl-diphosphate delta isomerase 2; ROCK, rho-associated coiled-coil kinase; RPE65, retinal pigment epithelium-specific 65; SCN7α, sodium channel protein type 7 subunit alpha; SOD, superoxide dismutase; SRXN1, sulfiredoxin 1; SIRT1, surtuin 1; TBHP, tert-Butyl hydroperoxide; TLR, toll-like receptor; TNF-α, tumor necrosis factor alpha; TRAF5, TNF receptor-associated factor 5; TRIB3, tribbles pseudokinase 3; TrxR1, thioredoxin reductase 1; TSPO, translocator protein; UVB, ultraviolet B; VEGF, vascular endothelial growth factor; Vom2r65, vomeronasal 2 receptor, 65; XBP1s, spliced X-box binding protein 1; xCT, Na^+^-independent-cysteine/glutamate exchanger; ZO-1, zonula occludens-1; γ-GCS, gamma-glutamylcysteine synthetase; γH2AX, phosphorylated histone H2AX; 8-OHdG, 8-hydroxydeoxyguanosine.

#### 4.7.1. Saffron

Saffron, the dried stigmas of *Crocus sativus* flowers, is a well-known spice which is highly valued for its golden color, flavor and aroma in the preparation of traditional dishes. A phytochemical analysis of saffron reported the presence of 150 compounds, of which carotenoids, crocin and crocetin were the most biologically active components [183]. Crocetin is a dicarboxylic carotenoid whereas crocin is a glycosylated carotenoid/crocetin.

Robust preclinical evidence established a strong rationale for testing the beneficial effect of long-term consumption of saffron in early-stage AMD. The studies involving a dietary saffron [176] and a patented saffron [182] show the key role of MMP-3 in the protection against photoreceptor apoptosis in animal models of light-induced retinal degeneration. Moreover, activation of ERK1/2 by crocetin has been found to demonstrate a protective role in a cellular model of Tert-butyl hydroperoxide-induced oxidative damage through the preservation of redox homeostasis and energy-yielding pathways [94]. Metabolic pathways such as glycolysis and mitochondrial respiration are the major sources of adenosine 5′-triphosphate (ATP) production. Indeed, nonspecific oxidative damage induced by excessive mitochondrial ROS production are observed together with increased protein aggregation and inflammation in AMD.

In a randomized, double-blinded, placebo-controlled crossover study, saffron supplementation for 90 days was found to improve macular function as assessed by focal electroretinogram (fERG) [184], accompanied by a significant increase in the average visual acuity on Snellen eye chart. The crossing over of AMD patients was conducted in accordance with the principles outlined by Maccarone et al. [185]. fERG is a tool for diagnosis, analysis of pathogenesis, prediction of prognosis and estimation of retinal flicker sensitivity in patients with early-stage AMD. A fERG can be recorded from the macular region in response to a continuous flickering of the straylight source in the periphery.

In addition, open-label longitudinal studies and double-blind randomized controlled trials have proven the efficacy of saffron in delaying the progression of dry [180], mild to moderate- [177,181] and advanced-stage AMD [179]. Di Marco et al. [182] postulated that saffron is superior to AREDS in delaying the progression of AMD. Considering that these findings were based on a relatively well-nourished American population, recommendations should be based on risk factors and demographic data.

Furthermore, common genetic variants, including complement factor H (CFH), complement factor I (CFI), complement factor B (CFB), complement 3 (C3) and human serine protease high temperature requirement A1 (HTRA1) [186], as well as the substitution of serine for alanine at codon 69 (A69S) in age-related maculopathy susceptibility 2 (ARMS2) gene positioned at a locus on chromosome 10q26 [187], are associated with AMD. Interestingly, Marangoni et al. [178] revealed that the functional effects of long-term saffron supplementation in improving visual function were not influenced by genetic polymorphisms associated with risk of AMD.

#### 4.7.2. Other Whole Foods

Organisciak et al. [128] studied the synergistic properties of an admixture of rosemary oil and zinc oxide (ZnO) in an animal model of light-induced retinal damage. The mineral supplementation was adjusted based on the recommendation of the Age-Related Eye Disease Study 1 (AREDS1) Research Group. The combination was found to be effective in preventing the progression of advanced-stage AMD by restoring the level of rhodopsin, cone opsin and cone arrestin, and decreasing the level of carboxyethylpyrrole (CEP) adducts. Furthermore, the inflammatory response was regulated by HO-1, a potent antioxidant. Cone phototransduction and survival are essential for color vision and visual acuity. As the rods and cones degenerate under a variety of pathological conditions including AMD, biomarkers are the most reliable prognostic indicators of disease progression. CEP adducts, derived from fragmentation of docosahexaenoate (DHA)-containing lipids, have been compellingly linked to AMD by triggering pathological angiogenesis.

Likewise, the antiangiogenic effects of defined grape powder [146], fermented *Capsicum annuum* or paprika [175] and rice bran [174] in apoptotic RPE cells that release VEGF were regulated through the inhibition of HIF and VEGF expression mediated by phosphoinositide 3-kinases (PI3K)/AKT and MAPK-dependent pathways.

**Figure 3:**
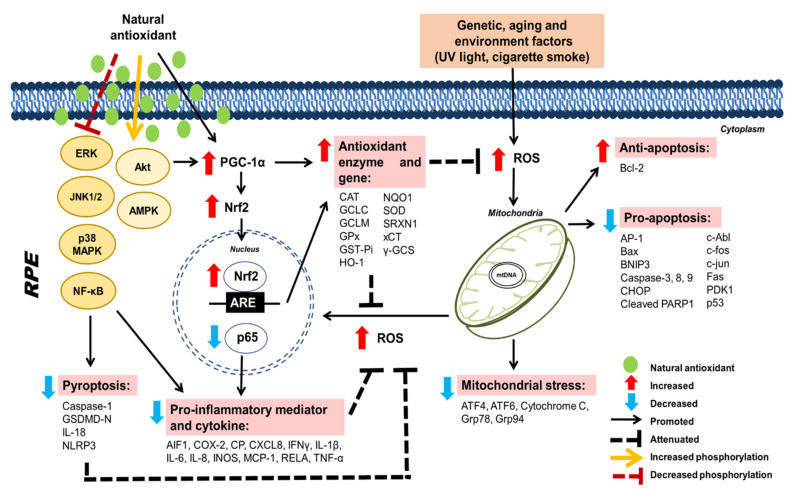
Genetic, aging and environmental factors exaggerate dysfunction and degeneration of retinal pigment epithelium (RPE) in age-related macular degeneration (AMD). Excessive production of reactive oxygen species (ROS) leads to mitochondrial dysfunction and mitochondrial DNA (mtDNA) damage, and therefore causing extracellular accumulation of insoluble protein aggregates. Modulation of extracellular-signal-regulated kinase (ERK), c-Jun N-terminal kinase (JNK)1/2, p38 mitogen-activated protein kinase (MAPK), nuclear factor kappa-light-chain-enhancer of activated B cells (NF-κB), protein kinase B (Akt) and adenosine monophosphate-activated protein kinase (AMPK) activities by natural antioxidants results in decreased pyroptosis, inflammation, mitochondrial stress and apoptotic activity. Upregulation of peroxisome proliferator-activated receptor gamma coactivator 1-alpha (PGC-1α) and nuclear factor erythroid 2-related factor 2 (Nrf2) promotes the expression of antioxidant genes and enhances the capacity of antioxidant defense systems in attenuating the damaging effects of ROS and reversing mitochondrial dysfunction. AIF1, allograft inflammatory factor 1; Akt, protein kinase B; AMPK, adenosine monophosphate-activated protein kinase; AP-1, activator protein-1; ARE, antioxidant response element; ATF, activating transcription factor; Bax, Bcl2 associated X; Bcl-2, B-cell lymphoma 2; BNIP3, BCL2 interacting protein 3; c-Abl, tyrosine-protein kinase ABL; CAT, catalase; CHOP, C/EBP homologous protein; COX-2, cyclooxygenase 2; CP, ceruloplasmin; CXCL8, C-X-C motif chemokine ligand 8; ERK, extracellular-signal-regulated kinase; GCLC, glutamate cysteine ligase catalytic subunit; GCLM, glutamyl cysteine ligase modifier subunit; GPx, glutathione peroxidase; Grp, glucose regulatory protein; GSDMD, gasdermin D; GSH, glutathione; GST-pi, glutathione S-transferase pi; HO-1, heme oxygenase-1; IFNγ, interferon gamma; IL, interleukin; INOS, inducible nitric oxide synthase; JNK, c-Jun N-terminal kinase; MAPK, p38 mitogen-activated protein kinase; MCP-1, monocyte chemoattractant protein-1; mtDNA, mitochondrial DNA; NF-κB, nuclear factor kappa-light-chain-enhancer of activated B cells; NLRP3, NLR family pyrin domain containing 3; NQO1, NAD(P)H dehydrogenase quinone 1; Nrf2, nuclear factor erythroid 2-related factor 2; PARP1, poly(ADP-ribose) polymerase 1; PDK1, pyruvate dehydrogenase kinase 1; PGC-1α peroxisome proliferator-activated receptor gamma coactivator 1-alpha; RELA, v-rel avian reticuloendotheliosis viral oncogene homolog A; ROS, reactive oxygen species; RPE, retinal pigment epithelium; SOD, superoxide dismutase; SRXN1, sulfiredoxin 1; TNF-α, tumor necrosis factor alpha; UV, ultraviolet; xCT, Na+-independent-cysteine/glutamate exchanger; γ-GCS, gamma-glutamylcysteine synthetase.

**Figure 4:**
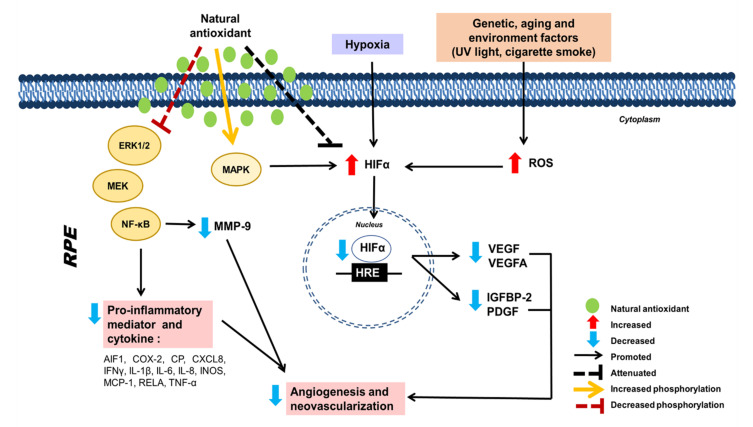
Hypoxia and excessive production of reactive oxygen species (ROS) promote active angiogenesis and neovascularization by upregulating mRNA expression of hypoxia-inducible factor-α (HIF-α). Modulation of extracellular-signal-regulated kinase (ERK)1/2, mitogen-activated protein kinase kinase (MEK), nuclear factor kappa-light-chain-enhancer of activated B cells (NF-κB) and p38 mitogen-activated protein kinase (MAPK) activities by natural antioxidants results in decreased inflammation and downregulation of hypoxia response element (HRE). Inhibition of vascular endothelial growth factor (VEGF) and platelet-derived growth factor (PDGF) lead to attenuation of angiogenesis and neovascularization. AIF1, allograft inflammatory factor 1; COX-2, cyclooxygenase 2; CP, ceruloplasmin; CXCL8, C-X-C motif chemokine ligand 8; ERK, extracellular-signal-regulated kinase; HIF-α, hypoxia-inducible factor-α; HRE, hypoxia response element; IFNγ, interferon gamma; IGFBP, insulin-like growth factor binding protein; IL, interleukin; INOS, inducible nitric oxide synthase; MAPK, p38 mitogen-activated protein kinase; MCP-1, monocyte chemoattractant protein-1; MEK, mitogen-activated protein kinase kinase; MMP, matrix metalloproteinase; NF-κB, nuclear factor kappa-light-chain-enhancer of activated B cells; PDGF, platelet-derived growth factor; RELA, v-rel avian reticuloendotheliosis viral oncogene homolog A; ROS, reactive oxygen species; RPE, retinal pigment epithelium; TNF-α, tumor necrosis factor alpha; UV, ultraviolet; VEGF, vascular endothelial growth factor.

Collectively, the effects of activating the signaling pathways which are responsible for antioxidant and anti-inflammatory-mediated effects and inhibiting VEGF of natural antioxidants, in terms of promoting the survival of RPE cells under oxidative damage, are shown in Figure 3 and Figure 4, respectively.

## 5. Limitations and Future Prospects

Although the results of this study are encouraging, several shortcomings are worth mentioning. Complementary and alternative medicines have been used for decades to treat various diseases including AMD [188,189]. Synergistic therapeutic effects have been demonstrated in herbal formulations and TCM [190,191]. The safety and effectiveness of some of these formulations, especially complementary and alternative medicines, are uncertain because no relevant, well-designed clinical trials or pharmacovigilance studies have been undertaken. Most of the prescribed formulations and herbal remedies used in primary health care have not been officially approved by the US Food and Drug Administration (FDA). Therefore, ophthalmologists should consider the potential risks and adverse effects before recommending these formulations to their patients [192].

In addition, the use of natural antioxidants, especially in traditional Chinese medicine, has been associated with some potential issues. A major challenge is the variation of raw compositions of natural antioxidants due to ecological and environmental differences, changing geography, phylogenetic cross-over, and species purity [193,194]. The therapeutic efficacy of plant-derived phytochemical compounds with high molecular weight is also of great concern due to their poor permeation through lipid bilayers and reduced bioavailability in humans [195]. The possible steroidal activity of TCM herbs and supplements in their raw and finished forms has been questioned, as they can lead to adverse reactions such as adrenal insufficiency, Cushing’s syndrome, hepatotoxicity, and nephrotoxicity. Some herbs could contain compounds that are structurally similar to steroids, which could interfere with steroid metabolism or bind to steroid receptors [196]. Therefore, analytical and high-throughput screening of the purified natural antioxidants is needed to understand the mechanisms of action of these natural products or traditional medicines [193].

Nevertheless, emerging trends in nanotechnology are revolutionizing the development of natural antioxidants. Nanotechnology can be used to facilitate the delivery of natural antioxidants and compounds by delaying the development of drug resistance, with improved responses comparable to modern medicine approaches [197]. In this respect, nanotherapeutics could be developed to facilitate the delivery of drug treatments for CNV by enhancing bioactivity, improving bioavailability at the target sites and allowing sustained drug release with prolonged action [198]. Although the use of nanomicellar and ophthalmic drop formulations may have therapeutic advantages for AMD [157,160], clinical trials are still needed to assess potential nanoparticle toxicity in the ocular surface, lens, retina and optic nerve.

In addition, the design of the study and choice of model are important for investigating the therapeutic effects and mechanisms of action of treatments targeting AMD. Primary cultures of human fetal and adult RPE cells are suitable for in vitro studies, as they retain the characteristics of native RPE tissue and are physiologically mature [199]. However, primary RPE cells cultured from different donors are genetically and physiologically different. Moreover, primary RPE cells tend to lose their RPE characteristics and their re-differentiation capacity after several passages [200]. Although there are a few commercially available continuous RPE cell lines, one limitation is that they lack their original pigmented phenotype, especially melanin [201,202]. Nevertheless, ARPE-19 cells can be repigmented using isolated melanosomes from porcine RPE, which promotes higher drug binding affinity and accumulation [203]. Furthermore, long-term appropriate culture and differentiation of low passage human ARPE cells can develop phenotypic characteristics and express genes specific to native RPE cells [204].

Although laser-induced CNV in animal models can mimic neovascularization with rapid CNV development, this is an acute injury and does not produce the complex chronic events leading to AMD. Injection-induced CNV can stimulate deposits and lesions, but this is less effective compared to laser-induced CNV. Transgenic murine models often exhibit symptoms of dry AMD, and thus, laser or injection is needed to stimulate neovascularization [199]. Laser-induced CNV in transgenic mice with expression or knock-out of gene variants can be used to test short-term hypotheses such as the involvement of environmental factors and external stress in AMD [205]. Moreover, rabbit models have the advantage of larger eyes, which is beneficial for the administration of subretinal injections and vectors for gene therapy [206]. However, laser-induced damage in the Bruch’s membrane and injection of proangiogenic factors are not applicable in rabbits, thus injection of Matrigel containing proangiogenic factors has been used to produce CNV lesions and disrupt Bruch’s membrane to resemble AMD [207].

Nonhuman primate animal models with mostly similar anatomy to human have also been used in preclinical studies. Unlike other animals, primates have a macula, which is needed for high acuity central vision and photoreceptor organization, and is an important part of the visual pathway [206,208]. Some nonprimate models are useful for AMD studies, including rhesus, cynomolgus and Japanese macaque monkeys that can have early-onset drusen, and African green monkeys that can have induced neovascularization by intravitreal injection of DL-alpha-aminoadipic acid [208,209,210]. Besides the difficulty in breeding and handling these animals, there are cost and ethical issues associated with their usage in AMD studies [211]. Before any animal study can be translated to a human study, it is crucial to consider the risk-benefit analysis, informed consent procedures, ethical review, and monitoring processes in order to develop a safe and effective treatment for AMD [212].

Current clinical studies on AMD, such as exploratory studies of gene variants, mRNA sequencing, and protein association, may not be sufficient to determine the cellular and signaling pathways. Consequently, different experimental designs, such as human clinical and pathological specimen analysis, imaging and genetic studies, mechanistic studies using in vitro and in vivo models (genetic, transcriptional, and proteomic studies) will be required [205]. Considering the prevalence of AMD is different among populations in different regions, the design of clinical studies should be standardized to allow consistent results for comparisons. A standardized grading system of AMD lesions should also be adopted in clinical studies such as the Wisconsin Age-Related Maculopathy Grading System, Age-Related Eye Disease Study System of Classifying AMD, the International Classification and Grading System for AMD, or the Clinical Classification of Age-Related Macular Degeneration [213]. Additionally, no validated clinical endpoints are accepted by all regulatory agencies. The only accepted primary endpoint in AMD clinical study is the best-corrected visual acuity (BVCA) that requires high contrast and high luminance, but it is not sensitive enough to detect the functional deficits in early or intermediate AMD [214,215]. Therefore, there is a need to develop and validate novel clinical endpoints or appropriate tools that are acceptable to regulatory agencies for clinical trials on early and intermediate AMD [216].

Generally, there has been a lack of animal and human studies evaluating the efficacy of natural antioxidants targeting AMD. Although there are many in vitro studies on the therapeutic impacts of curcumin, only one study of animal model of retinal disorder [217] and one retrospective study of AMD [91] have been revealed. One major disadvantage of retrospective studies is the small sample size, which may have selection bias, suboptimal handling of missing data and loss to follow-up. Although retrospective studies can reflect the results in the clinical setting compared to a randomized study, it does not accurately represent real world population outcomes [218,219]. Retrospective studies are also limited by their geographical reach, as the results cannot be applied to larger populations or other regions [220]. The signaling pathway of *Lycium barbarum* used in AMD is well elucidated in preclinical models. On the other hand, only two randomized controlled trials conducted in healthy older subjects and neovascular AMD patients have been reported [157,170]. Although randomized studies are often viewed as the gold standard for clinical evidence of the true relative efficacy of an intervention, there might be unknown or immeasurable confounding variables that can lead to a biased estimation of the treatment effect. Randomized studies are also time-consuming and expensive compared to other types of study [221].

In the future, a multitherapeutic approach should be adopted, as there is no experimental model that can emulate and represent the full pathophysiology of AMD. A model that is ideal for AMD research and the development of therapeutics should share similar anatomical features, physiological mechanisms and disease progression patterns with modifiable translational endpoints [222]. The “virtuous cycle” of bidirectional translation studies in AMD could be started by discovering and observing the human phenotype in AMD patients. Preclinical animal models could then be conducted to understand the pathophysiology and explore potential therapeutics. Finally, knowledge and potential therapeutics from preclinical studies need to be further tested and validated for their efficacy and tolerability in AMD patients [223]. Besides exploring other treatment options for wet AMD, there is a need to explore new treatments for dry AMD, as many potential therapeutic approaches targeting dry AMD have failed. Future therapeutic protocols will require treatments that target different aspects of AMD pathobiology, which will require concerted effort to discover potential therapies [224].

## 6. Conclusions

This review provides an overview of the available evidence from preclinical and clinical trials of the ability of natural antioxidants to improve or halt the progression of AMD. Nevertheless, there is increasing concern about the use of natural antioxidants, particularly traditional formulations, and their potential drug interactions. Elucidating the mechanisms underlying natural antioxidant-drug interactions also poses great challenges for AMD treatments. Another issue is the lack of adequate knowledge on the compositions and pharmacological actions of natural antioxidants. Therefore, scientific evidence and the dissemination of research are essential for the integration of natural antioxidants into evidence-based clinical practice.

## Figures and Tables

**Table 1:** Active compounds in the alleviation of AMD.

Active Compound	Model	Concentration/Dose	Finding	Mode of Action	Reference
Allicin 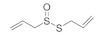	H_2_O_2_-induced oxidative damage in human ARPE-19 cell line	10–40 µg/mL	Protection against oxidative damage	↑ mRNA expression and protein level of Nrf2 ↑ SOD and NQO1 (antioxidant enzyme) ↓ mRNA expression and protein level of NOX4	[63]
Artemisinin 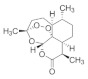	H_2_O_2_-induced oxidative damage in human D407 cell line and primary RPE cells	Various concentrations	Protection against oxidative damage and apoptosis	↑ pAMPKα	[64]
Astragaloside 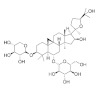	Isoflurane-induced apoptosis in primary RPE cells	50 µg/mL	Protection against apoptosis	↓ mRNA expression and protein level of CDC42, POLD1 and CCNA2 (cell cycle regulator), APH1B, APPBP2, NCSTN and APH1A (formation of β-amyloid), TRAF5 and NF-κB ↓ caspase-3/7	[65]
Berberine 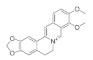	H_2_O_2_-induced oxidative damage in human D407 cell line and primary human RPE cells	1 and 3 µM	Protection against oxidative damage and apoptosis	↓ caspase-3/7 activation ↑ AMPK and total AMPK phosphorylation	[66]
	H_2_O_2_-induced oxidative damage in human D407 cell line and primary human RPE cells	Various concentrations	Protection against oxidative damage and apoptosis	↑ protein level of LC3B (autophagy marker) ↓ protein level of P62 (autophagy marker) ↑ AMPK and ULK1 phosphorylation ↓ mTOR phosphorylation	[67]
	LED light-induced retinal degeneration in BALB/c mice	200 mg/kg, PO	Protection against retinal degeneration	↑ mRNA expression of Rho, RPE65 and MCT3 ↓ mRNA expression of HMOX1, CP, CAT, GPx-1, SOD2 and AIF1 (oxidative damage and inflammatory marker)	[68]
Carnosic acid 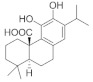	H_2_O_2_-induced oxidative damage in human ARPE-19 cell line and mouse photoreceptor-derived 661W cells	10 µM	Protection against oxidative damage	↑ mRNA expression and protein level of HO-1, NQO1, GCLM, xCT, NRF2 and SRXN1 (antioxidant enzyme) ↑ ARE activation and nuclear translocation of Nrf2 ↓ Prx2 hyperoxidation	[69]
	Light-induced retinal degeneration in Sprague-Dawley rats	25 mg/kg, IP	Protection against retinal degeneration	NE	
Celastrol 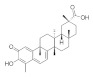	LPS-induced inflammation in human ARPE-19 cell line	0.05–1.5 µM	Protection against inflammation	↑ Hsp70 ↓ IL-6 and phosphorylated NF-κB p65 (pro-inflammatory cytokine)	[70]
Curcumin 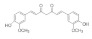	H_2_O_2_-induced-aging model in human ARPE-19 cell line	10–100 µM	Protection against oxidative damage and apoptosis	↑ Bcl-2 (anti-apoptotic protein) ↓ Bax and caspase-3 (pro-apoptotic protein)	[71]
	H_2_O_2_-induced oxidative damage in RPE cells derived from induced pluripotent stem cells (iPSCs) obtained from patients with dry AMD	10 μM	Protection against oxidative damage and apoptosis	↑ mRNA expression of HO-1, SOD2, and GPx1 (antioxidant enzyme) ↓ mRNA expression of PDGF, VEGF and IGFBP-2 (oxidative stress marker)	[72]
Curcuminoid Demethoxycurcumin 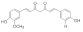 Bisdemethoxycurcumin 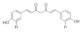	Blue light-induced cytotoxicity in human ARPE-19 cell line	15 μM	Protection against oxidative damage and apoptosis	↓ mRNA expression of c-Abl and p53 (pro-apoptotic factor)	[73]
Curcumin prodrug: Curcumin diethyl disuccinate 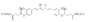	H_2_O_2_-induced oxidative damage in human ARPE-19 cell line	10 µM	Protection against oxidative damage	↑ mRNA expression and protein level of Bcl-2, and HO-1 and NQO1 (antioxidant enzyme) ↓ mRNA expression and protein level of phosphorylated p44/42 MAPK and Bax	[74]
Diarylheptanoid 7-(3,4 dihydroxyphenyl)-5-hydroxy-1-phenyl-(1E)-1-heptene 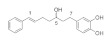	H_2_O_2_-induced oxidative damage in human ARPE-19 cell line	20 µM	Protection against oxidative damage and apoptosis	NE	[75]
Diphlorethohydroxycarmalol 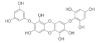	H_2_O_2_-induced oxidative damage in human ARPE-19 cell line	25 and 50 µM	Protection against oxidative damage and apoptosis	Modulation of γH2AX and 8-OHdG (DNA damage marker) ↑ pro-caspase-9 and pro-caspase-3 (anti-apoptotic protein ↓ cytochrome c, Bax and cleaved poly (ADP-ribose) polymerase (PARP) (pro-apoptotic protein)	[76]
FLZ 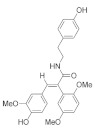	H_2_O_2_-induced oxidative damage in human ARPE-19 cell line and primary mouse RPE cells	1–25 µM	Protection against oxidative damage and apoptosis	↑ Akt activation	[77]
	TNF-α-induced inflammation in human ARPE-19 cell line	10–50 µg/mL	Protection against inflammation	↓ mRNA expression of ICAM-1 ↓ NF-κB p65 and phosphorylated IκBα	[78]
Ginsenoside 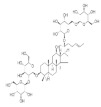	Human ARPE-19 cell line	250 nM	Combination of ginsenoside-Rb1 and VEGF reduced the secretion of VEGF	NE	[79]
	Human donor eyes	Various concentrations	Improvement of hydraulic and diffusional transport across Bruch’s membrane	NE	[80]
Glycyrrhizin 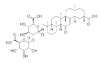	Sodium iodate-induced oxidative damage in human ARPE-19 cell line	20–200 µmol	Protection against oxidative damage and apoptosis	↑ p-Akt, Nrf2 and HO-1 ↓ cleaved caspase-3 (pro-apoptotic protein)	[81]
	Sodium iodate-induced retinal degeneration in C75BL/6 mice	50 mg/kg, IP	Protection against retinal apoptosis	NE	
GPETAFLR 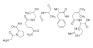	H_2_O_2_-induced oxidative damage in human ARPE-19 cell line	50 and 100 µg/mL	Protection against oxidative damage and inflammation	↓ mRNA expression and protein level of IL-1β, IL-6, TNF-α, IFNγ and VEGF (pro-inflammatory cytokine)	[82]
Gypenoside 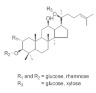	Oxidized low-density lipoprotein-induced oxidative damage in human ARPE-19 cell line	5 µg/mL	Protection against oxidative damage and inflammation	↑ mRNA expression and protein level of LXRα, TSPO, ABCA1, ABCG1, CYP27A1 and CYP46A1 (cholesterol metabolism and trafficking) ↓ NF-κB p65, IL-1β, IL-6, IL-8 and TNFα (inflammatory cytokine), and LDLR	[83]
Kinsenoside 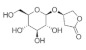	H_2_O_2_-induced oxidative damage in human ARPE-19 cell line	Various concentrations	Protection against oxidative damage and apoptosis Reduced VEGF secretion	↓ ERK and p38 phosphorylation, VEGF and NF-κB	[84]
Phillyrin 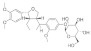	H_2_O_2_-induced oxidative damage in human ARPE-19 cell line	5–20 µM	Protection against oxidative damage and apoptosis	↑ Bcl-2, pro-caspase-8, pro-caspase-9 and pro-caspase-3 (anti-apoptotic protein), cyclin E, CDK2, cyclin A, total Nrf2 and nuclear Nrf2 ↓ Bax, cytochrome c and Fas (pro-apoptotic protein), p53, p-p53, p21 and Keap1	[85]
Rosmarinic acid 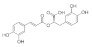	New Zealand white rabbits	400 µg, IV implant	Protection against retinal degeneration	NE	[86]
Total saponins Polyphyllin I 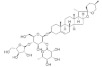 Polyphyllin II 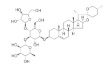 Polyphyllin VII 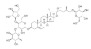 Polyphyllin H 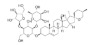	H_2_O_2_-induced oxidative damage in human ARPE-19 cell line	10–40 µg/mL	Protection against oxidative damage and apoptosis	↑ Bcl-2 (anti-apoptotic protein), Nrf2, HO-1, γ-GCS and NQO1 ↓ mRNA expression and protein level of Fas, FasL, Bax and caspase-3 (pro-apoptotic factor)	[87]

**Table 2:** Carotenoids in the alleviation of AMD.

Carotenoid	Model	Concentration/Dose	Finding	Mode of Action	Reference
β-cryptoxanthin 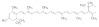	LED light-induced retinal degeneration in Wistar Albino rats	2 and 4 mg/kg, PO	Protection against oxidative damage	Modulation of ATF4, ATF6, Grp78, Grp94 (mitochondrial stress marker) ↑ Bax and caspase-3 (pro-apoptotic protein), HO-1 (antioxidant enzyme), NCAM and GAP-43 ↓ IL-1β, IL-6 and NF-KB (inflammatory cytokine), Bcl-2 (anti-apoptotic protein), GFAP and VEGF	[93]
Crocetin 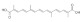	TBHP-induced oxidative damage in human ARPE-19 cell line	1–200 µM	Protection against oxidative damage	Preservation of energy production pathways ↑ ERK1/2 activation	[94]
Lutein and zeaxanthin Lutein 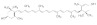 Zeaxanthin 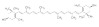	UVB irradiation-induced oxidative damage in human ARPE-19 cell line	5 µM	Protection against oxidative damage	↓ p38 MAPK and JNK1/2 phosphorylation	[95]
	Double-blind randomized controlled trial in young healthy subjects	10 mg/day lutein and 2 mg/day zeaxanthin	Increased serum levels of lutein and zeaxanthin; and macular pigment optical density Improvement in chromatic contrast and recovery from photostress	NE	[96]
Meso-zeaxanthin 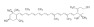	Double-blind randomized controlled trial in patients with non-advanced-stage AMD	10 mg meso-zeaxanthin in combination with co-antioxidants	Improvement in contrast sensitivity and visual function	NE	[97]
Undefined carotenoids	Prospective cohort study in healthy elderly subjects	Scoring of predicted plasma carotenoid	Long term reduced risk of developing advanced-stage AMD	NE	[98]

**Table 3:** Extracts and polysaccharides in the alleviation of AMD.

Extract/ Polysaccharide	Model	Concentration/Dose	Finding	Mode of Action	Reference
* Arctium lappa * ethanol extract	A2E-induced cytotoxicity in human ARPE-19 cell line	5–30 µg/mL	Protection against oxidative damage and apoptosis	↑ Bcl-2 (anti-apoptotic protein) ↓ Bax and cleaved caspase-3 (pro-apoptotic protein)	[106]
	White light-induced retinal degeneration in BALB/c mice	50–200 mg/kg, PO	Protection against retinal degeneration	NE	
Bilberry anthocyanin-rich aqueous extract	Light-induced retinal degeneration in pigmented rabbits	250 and 500 mg/kg, PO	Protection against photoreceptor apoptosis	↓ Bax, Bcl-2, and caspase-3 (pro-apoptotic protein); IL-1β and VEGF (inflammatory cytokine and angiogenic marker)	[107]
Bilberry ethanol extract	Blue light-emitting diode light-induced photoreceptor degeneration in murine photoreceptor (661 W) cells	10 µg/mL	Protection against oxidative damage	↓ LC3 autophagy marker), caspase-3/7 (pro-apoptotic protein), p38 MAPK and NF-_K_B activation	[108]
*Bucida buceras* ethanol extract	H_2_O_2_-induced oxidative damage in human ARPE-19 cell line	Various concentrations	Protection against oxidative damage and apoptosis	↓ caspase-3 (pro-apoptotic protein)	[109]
*Centella asiatica* ethanol extract	MNU-induced apoptosis in human RPE-19 cell line	Various concentrations	Protection against oxidative damage and apoptosis	↓ caspase-8, pro-caspase-9, pro-caspase-3 and pro-PARP (pro-apoptotic protein), p21 and CDK2	[110]
	Blue light-induced oxidative damage in human RPE cell line	Various concentrations	Protection against oxidative damage	NE	
	MNU-induced retinal degeneration in C57BL/6 mice	50–100 mg/kg, PO	Protection against retinal degeneration and apoptosis	↑ Nrf2 and HO-1 (antioxidant enzyme) ↓ caspase-3 and pro-caspase-3 (pro-apoptotic protein)	
Cranberry ethyl acetate extract	Blue light-induced oxidative damage in human ARPE-19 cell line	5–50 µg/mL	Protection against oxidative damage	NE	[72]
Crude fucoidan	TBHP-induced oxidative damage in human ARPE-19 cell line and primary RPE cells	1–250 µg/mL	Reduced VEGF secretion	NE	[111]
*Curcuma longa* ethanol extract	Blue light-induced cytotoxicity in human ARPE-19 cell line	15 μM	Protection against oxidative damage and apoptosis	↓ mRNA expression of c-Abl and p53 (pro-apoptotic factor)	[73]
*Diospyros kaki* ethanol extract	H_2_O_2_-induced oxidative damage in immortalized rat retinal precursor cell line (R28)	Various concentrations	Protection against oxidative damage	NE	[112]
	MNU-induced retinal degeneration in C57BL/6J mice	10–100 mg/kg, PO	Protection against retinal degeneration	↑ rhodopsin (retinal factor) ↓ nectin and GFAP (retinal factor), SOD1, SOD3 and GPx-1 (antioxidant enzyme)	
*Emblica officinalis* extract	Amyloid-β-induced cellular stress in human RPE AMD transmitochondrial cybrid cells	25 mg/mL	Protection against oxidative damage and apoptosis	↑ mRNA expression of MT-RNR2, SOD2 and PGC-1α ↓ caspase-3/7 (pro-apoptotic protein) ↓ mRNA expression of caspase-3 (pro-apoptotic factor) and VEGF (angiogenic marker)	[113]
Fucoidan 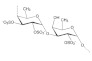	Human ARPE-19 cell line, primary porcine RPE cells, RPE/choroid perfusion organ culture	100 µg/mL	Combination of fucoidan and bevacizumab reduced the secretion of VEGF and angiogenesis	↓ VEGF165	[114]
	H_2_O_2_- and TBHP- induced oxidative damage in OMM-1 and human ARPE-19 cell lines, and primary porcine RPE cells	1–100 µg/mL	Reduced VEGF secretion	NE	[115]
	H_2_O_2_- and TBHP- induced oxidative damage in OMM-1 and human ARPE-19 cell lines, and primary porcine RPE cells	10 µg/mL	Reduced VEGF secretion	NE	[116]
	H_2_O_2_-induced oxidative damage in OMM-1 and human ARPE-19 cell lines	1–100 µg/mL	Reduced VEGF secretion	NE	[117]
* Garcinia cambogia * extract	CoCl_2_-induced HIF activation in murine retinal cone cell line (661W) and human ARPE-19 cell line	1 mg/mL	Protection against HIF activation	↓ mRNA expression and protein level of VEGFA, HIF-1α, BNIP3 and PDK1 (angiogenic marker and pro-apoptotic factor)	[118]
	Laser-induced CNV in C57BL6/J mice	0.2% extract mixed with MF diet, 30 mg/kg, IP	Protection against CNV	↓ HIF-1α	
Grape skin extract	Blue light-induced oxidative damage in human ARPE-19 cell line	0.2–5 µg/mL	Protection against A2E oxidation, apoptosis	↑ mRNA expression and protein level of GRP78 (ER stress and unfolded protein response marker); Bcl-2 (anti-apoptotic factor) ↓ CHOP, JNK, p-JNK, Bax, caspase-9, caspase-3, cleaved caspase-3 and cleaved caspase-9 (pro-apoptotic protein)	[119]
Lactoferrin 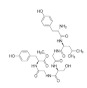	CoCl_2_-induced HIF activation in 661W and human ARPE-19 cell line	1 mg/mL	Protection against HIF activation	↓ mRNA expression of Pdk1, VEGFA and Glut1 (hypoxia response element)	[120]
	Laser-induced CNV in C57BL6/J mice and Hif1a conditional knockout mice	1600 mg/kg	Protection against CNV	↓ HIF-1α	
Lingonberry ethanol extract	Blue light-emitting diode light-induced photoreceptor degeneration in cultured murine photoreceptor (661 W) cells	10 µg/mL	Protection against oxidative damage	↓LC3 (autophagy marker), caspase-3/7 (pro-apoptotic protein), p38 MAPK and NF-KB activation	[108]
*Lycium barbarum* aqueous and ethanol extracts	UVB irradiation-induced growth arrest in human ARPE-19 cell line	25–50 μg/mL	Protection against DNA damage and apoptosis	↑ toll-like receptor (TLR), peroxisome proliferator-activated receptor (PPAR) and integrin activation	[121]
*Lycium barbarum* polysaccharides	H_2_O_2_-induced oxidative damage in human ARPE-19 cell line	10–5000 µg/mL	Protection against oxidative damage and apoptosis	↑ Bcl-2 ↓ Bax	[122]
	Aβ_1–40_ oligomers-induced retinal degeneration in human ARPE-19 cell line	3 and 14 mg/L	Protection against pyroptosis	↓ IL-1β, IL-18, NLRP3, caspase-1 and membrane GSDMD-N (pyroptosis-related proteins)	[123]
	Light-induced retinal degeneration in BALB/cJ mice	150 and 300 mg/kg, PO	Protection against photoreceptor degeneration	↑ mRNA expression of Nrf2 and TrxR1 ↓ mRNA expression of PARP14	[124]
* Melissa officinalis * ethanol extract	H_2_O_2_-induced oxidative damage in human ARPE-19 cell line	100 µg/mL	Protection against oxidative damage and apoptosis	↑ Akt phosphorylation ↓ caspase-3/7 and PARP cleavage (pro-apoptotic protein)	[125]
*Pueraria lobata* ethanol extract	H_2_O_2_-induced oxidative damage in human ARPE-19 cell line	Various concentrations	Protection against oxidative damage	↑ ZO-1 ↓ p38 MAPK and JNK phosphorylation	[126]
Red wine extract	Human ARPE-19 cell line	30–100 µg/mL	Inhibition of VEGF-A secretion	↓ VEGF, VEGF-A, VEGF-R2 and phosphorylated VEGF-R2 (angiogenic marker); MEK and ERK ½ phosphorylation	[127]
Rosemary extract	White light-induced retinal degeneration in Sprague-Dawley rats	Various concentrations, IP	Protection against retinal degeneration	↑ HO-1 (antioxidant enzyme), rhodopsin, cone opsin, cone arrestin, retinal DNA and GFAP ↓ CEP (AMD biomarker)	[128]
Saudi * Origanum vulgare * extract-mediated gold nanoparticles	H_2_O_2_-induced oxidative damage in human RPE-19 cell line and human umbilical vein endothelial cells (HUVEC) and human RPE cells	0.1–1 mg/mL	Protection against oxidative damage and apoptosis	↓ mRNA expression of IL-6, TNF-α, caspase-3 and NLRP-3 (inflammatory cytokine and pro-apoptotic factor) ↓ VEGF and F4/80	[129]
* Solanum melongena * ethanol extract	Blue light-induced oxidative damage in human RPE cell line	Various concentrations	Protection against oxidative damage	↓ nuclear p65, CXCL8, IL-1β, RELA and PARP cleavage (inflammatory cytokine and pro-apoptotic protein) and NF-κB activation ↓ mRNA expression of CXCL8, NFKBIA, IL-1β, RELA, TRIB3 and XBPIs (inflammatory cytokine and unfolded protein response marker)	[130]
	Blue light-induced retinal degeneration in BALB/c mice	100 and 200 mg/kg, PO	Protection against retinal degeneration	NE	
*Tribulus terrestris* ethanol extract	H_2_O_2_-induced oxidative damage in human RPE-19 cell line	100 and 200 µg/mL	Protection against oxidative damage and apoptosis	↑ mRNA expression of Nrf2, CAT, SOD1, SOD2, GST-pi, HO-1, NQO1 and GCLM ↑ Bcl-2 (anti-apoptotic factor) and Nrf2 activation ↓ Bax, cleaved caspase-3 and cleaved caspase-9 (pro-apoptotic protein)	[131]
*Vaccinium uliginosum* water extract	Blue light-induced cytotoxicity in human ARPE-19 cell line	Various concentrations	Protection against oxidative damage and apoptosis	↓ caspase-3 and Bax/Bcl-2 ratio (pro-apoptotic protein)	[132]
	Blue light-induced cytotoxicity in human ARPE-19 cell line	Various concentrations	Protection against oxidative damage	NE	[133]
	Blue light-induced retinal degeneration in BALB/c mice	25, 50 and 100 mg/kg, PO	Protection against retinal degeneration	NE	

**Table 4:** Flavonoids in the alleviation of AMD.

Flavonoid	Model	Concentration/Dose	Finding	Mode of Action	Reference
Anthocyanin Cyanidin-3-O-glucoside 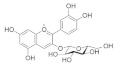 Malvidin 3-glucoside 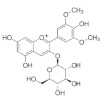 Malvidin 3-galactoside 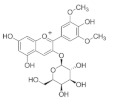	UVB irradiation-induced oxidative damage in human ARPE-19 cell line	5 µM	Protection against oxidative damage	↓ JNK1/2 and p38 MAPK phosphorylation	[95]
	H_2_O_2_-induced oxidative damage in human ARPE-19 cell line	5 µg/mL	Protection against oxidative damage and apoptosis	↑ Akt phosphorylation and Bcl-2 ↓ Erk1/2 and p38 phosphorylation; caspase-3 and Bax (pro-apoptotic protein) and VEGF	[135]
Chlorogenic acid 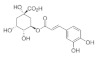	Light-induced retinal degeneration in pigmented rabbits	39.42 mg/kg, PO	Protection against retinal inflammation	↓ NF-κB activation	[136]
Epigallocatechin gallate 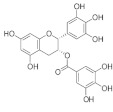	H_2_O_2_-induced oxidative damage in human ARPE-19 cell line	1–50 µM	Protection against ocular neovascularization and vascular permeability	↓ mRNA expression and protein level of MMP-9, VEGF, VEGF receptor-2 and TNF-α	[137]
	VEGF-induced vascular leakage in Sprague-Dawley rats Alkali burn-induced corneal angiogenesis in BALB/c mice	200 mg/kg, PO	Protection against ocular neovascularization and vascular permeability	↑ MMP-9 and platelet endothelial cell adhesion molecule (PECAM/CD31) ↓ vascular leakage and permeability	
Fisetin 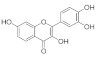	Etoposide-induced apoptosis in human ARPE cell line and primary human RPE cells	50 µM	Protection against inflammation	↓ IL-8 and IL-6 (inflammatory cytokine)	[138]
Homoisoflavonoids 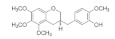	Human retinal microvascular endothelial cells (HRECs)	0.01–10 nM	Protection against angiogenesis	NE	[139]
Kaempferol 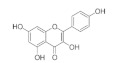	H_2_O_2_-induced oxidative damage in human ARPE-19 cell line	20 and 50 nM	Protection against oxidative damage and apoptosis	↑ mRNA expression and protein level of Bcl-2 (anti-apoptotic factor) ↓ mRNA expression and protein level of Bax and caspase-3 (pro-apoptotic factor)	[140]
	Sodium iodate-induced retinal degeneration in Sprague-Dawley rats	3%, intravitreal, IV	Protection against retinal degeneration and apoptosis	↑ RPE65 ↓ mRNA expression and protein level of VEGF	
Luteolin 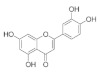	Etoposide-induced apoptosis in human ARPE cell line and primary human RPE cells	50 µM	Protection against inflammation	↓ IL-8 and IL-6 (inflammatory cytokine)	[138]
Proanthocyanidins 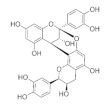	Light-induced retinal degeneration in Sprague-Dawley rats	30–300 mg/kg, PO	Protection against oxidative damage and apoptosis	NE	[141]
Quercetin 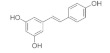	H_2_O_2_-induced oxidative damage in human ARPE-19 cell line	100 µM	Protection against oxidative damage and inflammation	↑ mRNA expression of Nrf2 and HO-1 ↓ mRNA expression of IL-6 and IL-1β (inflammatory cytokine)	[142]
	4-Hydroxynonenal-induced oxidative damage in human ARPE-19 cell line	50 µM	Protection against inflammation	↓ mRNA expression and protein level of IL-6, IL-8 and MCP-1 (inflammatory cytokine) ↓ p38, MAPK, ERK and CREB phosphorylation	[143]
	Light-induced retinal degeneration in pigmented rabbits	33.63 mg/kg, PO	Protection against oxidative damage and inflammation	↑ HO-1 (antioxidant enzyme) ↓ MCP-1, IL-8, IL-1β, TNF-α and COX-2 (inflammatory cytokine)	[136]
	Light-induced retinal degeneration in Sprague-Dawley rats	50 mg/kg, IP	Protection against photoreceptor apoptosis and retinal degeneration	↓ AP-1-regulated c-Jun/c-Fos heterodimerization	[144]
Resveratrol 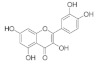	Human ARPE-19 cell line	100 µM	Combination of resveratrol and bevacizumab reduced the secretion of VEGF	↑ mRNA expression of Notch 4 ↓ MEK1/2 (Ser217/221) and 44/42 MAPK (Thr202/Tyr204) phosphorylation and vimentin	[145]
	Immorto mice (H-2K(b)-ts-A58(+/+) derived-choroidal endothelial cells	100 µM	Protection against CNV	↑ p53 (pro-apoptotic protein) ↓ Akt activation	[146]
	Hydroquinone-induced oxidative damage in primary human RPE cells	15 and 30 µM	Protection against oxidative damage	↑ mRNA expression and protein level of HO-1 and GCLC (antioxidant enzyme) ↓ XBP1	[147]

**Table 5:** Formulations in the alleviation of AMD.

Formulation	Model	Concentration/Dose	Finding	Mode of Action	Reference
AREDS and rosemary/carnosic acid/ursolic acid	Light-induced retinal degeneration in Sprague-Dawley rats	17 mg/kg, IP	Protection against retinal degeneration	↑ mRNA expression of EGR1, GNG11, RGD1564999, SCN7A, Olr425, Vom2r65, OPRK (retinal factor) ↑ HO-1 antioxidant enzyme) rhodopsin, rod S-antigen, cone opsin and cone arrestin ↓ CEP (AMD biomarker)	[152]
AREDS2	Double-blind randomized controlled trial in healthy elderly subjects	Dose-ranging, PO	No effect on reducing the risk of progression to advanced AMD	NE	[104]
Chuanqi microemulsion in situ gel	Sodium iodide-induced retinal degeneration in Sprague Dawley rats	20 μL, dripping	Protection against retinal degeneration	NE	[153]
Curcumin supplement	Retrospective case-control study in patients with neovascular AMD	NE	Combination of curcuma and anti-VEGF reduced the frequency of injections	NE	[91]
Fufang Xueshuantong	Prospective randomized controlled pilot study in patients with CNV	4500 mg/day, PO	Combination of Fufang Xueshuantong with ranibizumab reduced the CNV-PED complex thickness Improvement in BCVA	NE	[154]
Liquid formulation of omega-3 concentrate	Open-label pilot study in patients with dry AMD	3.4g of eicosapentaenoic acid (EPA) and 1.6g of docosahexaenoic acid (DHA), PO	Improvement in vision	NE	[155]
Lutein formulation	Light-induced retinal degeneration in Sprague-Dawley rats	104 mg/kg, PO	Protection against photoreceptor apoptosis and retinal degeneration	NE	[156]
Milk-based formulation of *Lycium barbarum*	Double-blind randomized controlled trial in healthy elderly subjects	13.7 g, PO	Protection against macula hypopigmentation and accumulation of soft drusen	NE	[157]
Mingjing	Double-blind randomized controlled trial in patients with neovascular AMD	5.95 g, PO	Combination of Mingjing and ranibizumab reduced the frequency of injections	NE	[158]
Nanomicellar drop	H_2_O_2_-induced oxidative damage in human D407 cell line	10 µM	Protection against oxidative damage	↓ VEGF	[159]
Ophthalmic drop formulation	Laser radiation-induced in a nonhuman primate model of AMD-rhesus monkey	1 mg/mL, dripping	Promotion of autophagy and suppression of angiogenesis	↑ 1,25D3-MARRS	[160]
Pharmaceutical composition (Patent No: WO2012079419)	Light-induced retinal CNV in Brown Norway rats	NA	Protection against CNV	NE	[161]
	Clinical trial in patients with neovascular CNV	NA	Protection against CNV	NE	
RESVEGA^®^	Human ARPE-19 cell line	Various concentrations	Inhibition of VEGF-A secretion	↓ VEGF-R2/Cav-1 complex dissociation into lipid rafts, and MAPK activation	[162]
Resveratrol formulation	Human RPE AMD transmitochondrial cybrid cells	1000 µM	Protection against oxidative damage	NE	[163]
Shihu Yeguang	Bright light-induced photoreceptor degeneration in BALB/c mice	57 mg/20 g, PO	Protection against retinal degeneration and apoptosis	↑ Bcl-2 (anti-apoptotic factor) ↓ mRNA expression of c-fos and c-jun (pro-apoptotic factor); TNF-α (pro-inflammatory cytokine)	[164]
Triphala	TNF-α-induced angiogenesis and inflammation in rhesus monkey choroidal-retinal endothelial cell line (RF/6A)	Various concentrations	Protection against inflammation, tube formation, chemotaxis and proliferation	↑ IL-10 and IL-13 (inflammatory cytokine) ↓ MMP-9; p38, ERK and NF-κB phosphorylation ↓ mRNA expression of IL-6, IL-8, eotaxin, MCP-1, MIP-1β, RANTES, IL-5 and PDGF-BB (inflammatory cytokine)	[165]
ZQMT	Randomized clinical trial in patients with CNV	15 tablets, PO	Improvement in visual acuity Combination of ZQMT and ranibizumab reduced the frequency of injections	NE	[166]
	Laser-induced CNV in Crb1rd8 mice	25 mg/mL, PO	Protection against AMD-related retinopathy	↑ CCL2 and CX3CR1 (chemokine axis) activation	[167]

**Table 6:** Vitamin in the alleviation of AMD.

Vitamin	Model	Concentration/Dose	Finding	Mode of Action	Reference
Vitamin B6 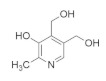	CoCl_2_-induced hypoxic condition in mouse photoreceptor-derived 661W and human ARPE-19 cell lines	1 mg/mL	Suppression of retinal neovascularization	↓ mRNA expression of VEGF ↓ HIF	[174]
	Light-induced retinal degeneration in C57BL/6 and BALB/c mice	9 and 35 mg/kg, PO	Suppression of retinal neovascularization	↓ HIF	

**Table 7:** Whole foods in the alleviation of AMD.

Whole Food	Model	Concentration/Dose	Finding	Mode of Action	Reference
Defined grape powder	Laser-induced CNV in C57BL/6J mice	100 mg/animal, PO	Protection against CNV	NE	[146]
Fermented *Capsicum annuum*	Sodium iodate-induced oxidative damage in human ARPE-19 cell line	500 µg/mL	Protection against oxidative damage and apoptosis	↓ cleaved PARP-1, caspase-8 and caspase-3 (pro-apoptotic factor); AKT, JNK and p38 phosphorylation	[175]
	Sodium iodate-induced retinal degeneration in C57BL/6 mice	195 mg/kg, PO	Protection against retinal degeneration		
*Lycium barbarum*	Double-blind randomized controlled trial in patients with neovascular AMD	25 g/day, PO	Improvement in macular pigment optical density	NE	[170]
Rice bran	CoCl_2_-induced hypoxic condition in mouse photoreceptor-derived 661W and human ARPE-19 cell lines	1 mg/mL	Suppression of retinal neovascularization	↓ HIF ↓ mRNA expression of VEGF	[174]
	Light-induced retinal degeneration in C57BL/6 and BALB/c mice	587.5 mg/kg, PO	Suppression of retinal neovascularization	↓ HIF	
Rosemary oil	White light-induced retinal degeneration in Sprague-Dawley rats	Various concentrations, IP	Protection against retinal degeneration	↑ HO-1 (antioxidant enzyme), rhodopsin, cone opsin, cone arrestin, retinal DNA and GFAP ↓ CEP (AMD biomarker)	[128]
Saffron	Light-induced retinal degeneration in Sprague-Dawley rats	1 mg/kg, PO	Protection against photoreceptor degeneration	NE	[176]
	Open-label longitudinal study in patients with AMD	20 mg/day, PO	Improvement in macular function in early/moderate-stage AMD	NE	[177]
	Open-label longitudinal study in patients with AMD	20 mg/day, PO	Improvement in macular function	NE	[178]
	Double-blind randomized controlled trial in patients with AMD	30 mg/day, PO	Improvement in retinal function in advanced-stage AMD	NE	[179]
	Clinical trial in patients with dry AMD	50 mg/day, PO	Improvement in visual function Delaying the progression of dry AMD	NE	[180]
	Double-blind randomized controlled trial in patients with AMD	20 mg/day, PO	Preservation of retinal function in mild/moderate-stage AMD	NE	[181]
Saffron (Patent: W02015/145316)	Light-induced retinal degeneration in albino rats	1 mg/kg	Protection against photoreceptor apoptosis and retinal degeneration	↓MMP-3	[182]
	Clinical trial in patients with AMD	NE	Delaying the progression of AMD	NE	

## Data Availability

Data sharing not applicable.
